# Milking the Cow: Cattle-Derived Chimeric Ultralong CDR-H3 Antibodies and Their Engineered CDR-H3-Only Knobbody Counterparts Targeting Epidermal Growth Factor Receptor Elicit Potent NK Cell-Mediated Cytotoxicity

**DOI:** 10.3389/fimmu.2021.742418

**Published:** 2021-10-25

**Authors:** Lukas Pekar, Daniel Klewinghaus, Paul Arras, Stefania C. Carrara, Julia Harwardt, Simon Krah, Desislava Yanakieva, Lars Toleikis, Vaughn V. Smider, Harald Kolmar, Stefan Zielonka

**Affiliations:** ^1^ Protein Engineering and Antibody Technologies, Merck Healthcare KGaA, Darmstadt, Germany; ^2^ Institute for Organic Chemistry and Biochemistry, Technische Universität Darmstadt, Darmstadt, Germany; ^3^ The Applied Biomedical Science Institute, San Diego, CA, United States; ^4^ Department of Molecular Medicine, The Scripps Research Institute, La Jolla, CA, United States

**Keywords:** antibody display, antibody engineering, cattle antibody, ultralong CDR3, yeast surface display, Knobbody

## Abstract

In this work, we have generated epidermal growth factor receptor (EGFR)-specific cattle-derived ultralong CDR-H3 antibodies by combining cattle immunization with yeast surface display. After immunization, ultralong CDR-H3 regions were specifically amplified and grafted onto an IGHV1-7 scaffold by homologous recombination to facilitate Fab display. Antigen-specific clones were readily obtained by fluorescence-activated cell sorting (FACS) and reformatted as chimeric antibodies. Binning experiments revealed epitope targeting of domains I, II, and IV of EGFR with none of the generated binders competing with Cetuximab, Matuzumab, or EGF for binding to EGFR. Cattle-derived chimeric antibodies were potent in inducing antibody-dependent cell-mediated cytotoxicity (ADCC) against EGFR-overexpressing tumor cells with potencies (EC_50_ killing) in the picomolar range. Moreover, most of the antibodies were able to significantly inhibit EGFR-mediated downstream signaling. Furthermore, we demonstrate that a minor fraction of CDR-H3 knobs derived from generated antibodies was capable of independently functioning as a paratope facilitating EGFR binding when grafted onto the Fc part of human IgG1. Besides slightly to moderately diminished capacities, these engineered Knobbodies largely retained main properties of their parental antibodies such as cellular binding and triggering of ADCC. Hence, Knobbodies might emerge as promising tools for biotechnological applications upon further optimization.

## Introduction

Antibody therapeutics have been proven to be of utmost relevance for the treatment of life-threatening conditions, for instance, oncological or infectious diseases and immunological disorders. Today, approximately 100 antibody derivatives have been granted marketing approval by the Food and Drug Administration (FDA) with more than 830 entities in clinical development (1, 2). It is projected that by the year 2025, the global antibody sector will be valued 300 billion US dollars (3). As such, antibodies will represent one of the main drivers of the pharmaceutical sector.

In addition to canonical antibodies composed of heavy and light chains, the adaptive immune system of several species such as camelids or sharks produces non-conventional scaffolds, for instance, heavy-chain only antibodies that display several beneficial attributes with respect to biomedical applications (4–6). Moreover, also non-immunoglobulin-based humoral components have been identified that respond to foreign antigen in an adaptive manner (7–9).

It has been known since the late 1990s that a subset of bovine antibodies displays a peculiarly long CDR-H3 region (10, 11) with up to 70 amino acids forming a protruding paratope (12). Interestingly, the vast majority of bovine antibodies harboring ultralong CDR-H3 regions is composed of one distinct V gene segment, IGHV1-7 (also referred to as VH_BUL_), and one distinct D gene segment, IGHD8-2. Moreover, it appears that these ultralong heavy chains preferably pair with λ light chains of the V30 segment (12, 13). Due to the low somatic variability of IGHV1-7 and the restricted utilization of light chains, there is some evidence that antigen binding almost exclusively depends on CDR-H3 with other regions within the variable domains having a stabilizing function (12, 14). From a structural perspective, nearly all known ultralong CDR-H3 regions adopt a similar structure that can be divided into a stalk region composed of an ascending and a descending β-strand and the knob region, solely encoded by the D segment IGHD8-2. This germline D segment naturally harbors four cysteines (12, 14). In addition, 38 codons within IGHD8-2 can be readily mutated to cysteine *via* only one nucleotide exchange by a diversification process involving activation-induced cytidine deaminase (12), resulting in an unprecedented structural diversity of the knob region mediated by different disulfide bond patterns (15). Intriguingly, also nature-derived cysteine-rich miniproteins have been engineered for a plethora of applications (16–18) culminating in FDA-approved therapeutic entities Ziconotide and Linaclotide (19, 20). Due to their structural peculiarities, bovine ultralong CDR-H3 antibodies have been harnessed to target different antigenic components, with most of them being related to infectious diseases such as HIV (21–23). Furthermore, cow antibodies have been developed targeting complement component C5 (24, 25). In the same work, the authors were able to show that the bovine knob domain can function autonomously, albeit the knob domains were generated in a complex process involving expression as Fab fragment followed by tobacco etch virus (TEV)-mediated cleavage and release of the knob domain.

**Figure 1:**
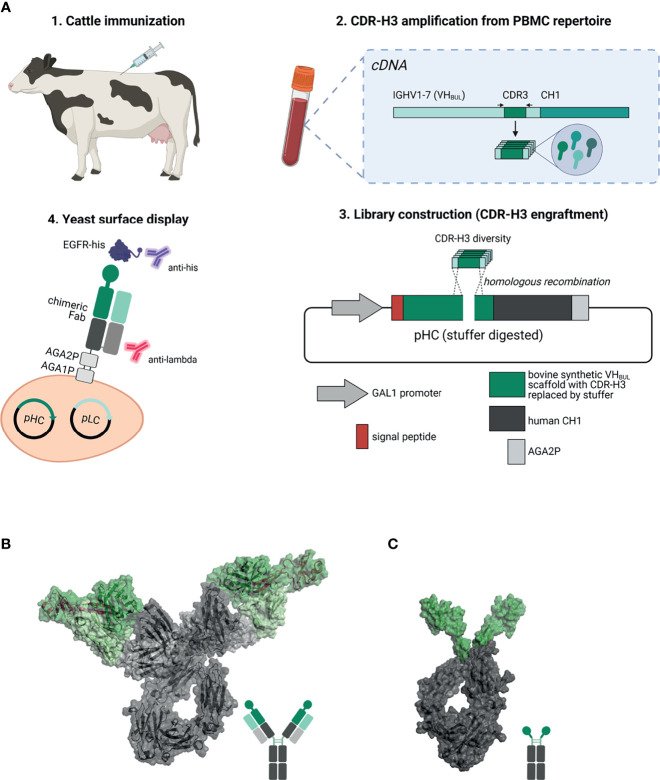
Overview about the generation of EGFR-specific ultralong CDR-H3 antibodies and Knobbodies. **(A)** Scheme of the process of isolating cattle-derived ultralong CDR-H3 Fab fragments. (1.) Cattle were immunized with recombinant human EGFR. After immunization and blood collection, peripheral blood mononuclear cells (PBMCs) were isolated followed by RNA extraction and cDNA synthesis. (2.) Ultralong CDR-H3 regions were specifically amplified from generated cDNA. (3.) Amplified CDR-H3 regions were inserted into a display plasmid harboring a synthetic VH_BUL_ domain fused to human CH1 and AGA2P by homologous recombination. (4.) Yeast surface display was utilized to select for EGFR-targeting chimeric Fab fragments. Dark green: synthetic cattle derived IGHV1-7 scaffold with engrafted stalk/knob diversity. Light green: Bovine VL30 region. Dark gray: human CH1 domain. Light gray: human CLλ region. Resulting EGFR-specific paratopes were reformatted as chimeric IgG antibodies **(B)** and knob-human Fc fusion proteins referred to as Knobbodies **(C)**. Cattle-derived VH1-7 domain encompassing engrafted CDR-H3 diversity encoding for stalk and knob architectures shown in dark green and bovine VL30 region colored in light green. Knob and stalk structures colored in purple in the cartoon structure. Human IgG1 backbone colored in dark gray, human CLλ colored in light gray **(B)**. Knob region shown in dark green as grafted onto a human IgG1 backbone colored in dark gray **(C)**. Model generated using PYMOL v0.99 based on pdb entries 5dk3 and 5ilt. Schemes generated with biorender (www.biorender.com).

In this study, we have established a platform process for facile generation of ultralong CDR-H3 antibodies following cattle immunization. To this end, stalk-knob comprising CDR-H3 regions were specifically amplified and grafted onto a synthetic (IGHV1-7 and LC V30 derived) chimeric Fab scaffold for yeast surface display (YSD, **Figure 1A**). We were able to readily isolate ultralong CDR-H3 antibodies targeting cancer-associated receptor tyrosine kinase epidermal growth factor receptor (EGFR) (26, 27). After reformatting into chimeric antibody backbones (**Figure 1B**), isolated binders exhibited specific binding to EGFR with a wide range of affinities and targeting of three different domains on EGFR, demonstrating a rather broad epitope coverage. Moreover, specific cellular binding properties on EGFR-expressing tumor cells were observed. Interestingly, albeit not competing with epidermal growth factor (EGF) for EGFR binding, the majority of clones inhibited EGFR-dependent signal transduction as demonstrated by AKT phosphorylation. In addition, all generated variants triggered significant ADCC with two variants displaying similar capacities to therapeutic antibody Cetuximab (28).

Furthermore, we also constructed knob-Fc fusion proteins by grafting the knob region without the stalk of EGFR-specific CDR-H3 regions onto the hinge region of human IgG1 (**Figure 1C**). Most of the resulting fusion proteins showed propensities for aggregation. Intriguingly, two resulting knob-Fc fusion proteins, referred to as Knobbodies, displayed acceptable biophysical properties as indicated by expression yields and target monomer levels in size exclusion chromatography (SEC). Most importantly, main functionalities such as cellular binding, antagonism, or ADCC capacities of their parental antibodies were largely retained while being substantially smaller in size. Essentially, cattle-derived ultra-long CDR-H3 antibodies and engineered knob domains might open up new avenues in terms of engineering next generation antibody entities upon further optimization.

## Material and Methods

### Cattle Immunization

Three cattle (*Bos taurus*) with ages between 9 months and 11 months were immunized with recombinant human EGFR (extracellular domain containing *C*-terminal hexahistidine- tag, produced and purified in-house) as cocktail approach with unrelated antigen at preclinics GmbH. All experimental procedures and animal care were in accordance with local animal welfare protection laws and regulations [Niedersächsisches Landesamt für Verbraucherschutz und Lebensmittelsicherheit (LAVES), Dezernat 33—Tierschutzdienst, number 33.19-42502-05-17A210]. In brief, for each immunization, 200 µg of EGFR diluted in a volume of 2 ml were mixed with 2 ml of Fama adjuvant (GERBU Biotechnik) and administered subcutaneously at multiple sites. In total, six immunizations were performed over the course of 84 days (d0, d28, d42, d56, d70, and d84). On day 88, blood (250 ml per specimen) was collected, and total RNA was extracted, followed by complementary DNA (cDNA) synthesis. To monitor the immune response, a direct ELISA was conducted. In short, recombinant human EGFR (5 µg/ml) was coated onto 96-well plates overnight at 4°C. After washing with phosphate-buffered saline (PBS) and blocking with PBS/bovine serum albumin (BSA) for 60 min at room temperature (RT), serum samples in different dilutions (in PBS/BSA) were added in a volume of 25 µl for 60 min at RT. After washing, titer was detected using anti-bovine IgG (H&L specific) horseradish peroxidase (HRP) conjugate, diluted 1:25,000 in PBS/BSA and TMB One solution and 1 M sulfuric acid.

### Strains, Media, and Plasmids

For CDR-H3 (VH) library generation and for epitope mapping, *Saccharomyces cerevisiae* strain EBY100 [*MATa URA3-52 trp1 leu2Δ1 his3Δ200 pep4::HIS3 prb1Δ1.6R can1 GAL (pIU211:URA3*)] was employed (Thermo Fisher Scientific). For light chain construction, strain BJ5464 (*MATα URA3-52 trp1 leu2Δ1his3Δ200 pep4::HIS3 prb1Δ1.6R can1 GAL*) (American Type Culture Collection) was used. Yeast cells were cultivated in media as described elsewhere (29). Genetic elements of plasmids were deduced from pYD1 plasmid backbone (Yeast Display Vector Kit, version D, #V835-01, Thermo Fisher Scientific). Light chain plasmid pLC contained leucine marker and kanamycin resistance genes and encoded for αMFpp8 signal sequence, a stuffer sequence (with *Bam*HI and *Not*I cleavage sites enabling VL cloning by homologous recombination) followed by the human constant lambda region. Expression was facilitated by galactose inducible promoter (*GAL1*). The heavy chain plasmid (pHC) comprised a tryptophan auxotrophic marker and an ampicillin resistance marker. pHC encoded for the *AGA2* signal peptide, followed by a synthetic bovine IGHV1-7 domain fused to human constant region CH1 and AGA2P. CDR-H3 was replaced by a stuffer sequence harboring *Not*I and *Eco*RI enabling engraftments of PCR amplified CDR-H3 regions by gap repair cloning (**Supplementary Figure S2**). Expression was also under the control of the *GAL1* promoter.

### Yeast Surface Display Library Construction

Bovine ultralong CDR-H3 regions were amplified from cDNA using three forward primers and two reverse primers specifically targeting FR3 of IGHV1-7 and FR4 and concomitantly incorporating overhangs for homologous recombination in *S. cerevisiae* (**Supplementary Table S1**). The PCR was conducted using 16 reactions in parallel for each primer set in a total volume of 50 µl using Q5 High-Fidelity 2× Master Mix (New England Biolabs; NEB) and 1 µl cDNA pooled from three different bovine specimen. Conditions for CDR-H3 amplification were as follows: 98°C for 3 min for initial denaturation, 35 cycles of 30 s at 98°C and 50 s at 72°C, followed by 2 min at 72°C. For amplification of the single light chain (VLλ30) used in this study, a gene string (encoding for the light chain, synthesized at GeneArt, Thermo Fisher Scientific) was used as template (20 ng) with a fixed primer set (**Supplementary Table S1**) and High-Fidelity 2× Master Mix in a volume of 50 µl. PCR was performed at 98°C for 30 s, 35 cycles of 98°C for 10 s, 62°C for 20 s, and 72°C for 20 s, followed by 2 min at 72°C. PCR products were pooled for each primer set and purified using Wizard^®^ SV Gel and PCR Clean-up System (Promega). Destination plasmids for CDR-H3 (pHC) and VL (pLC) cloning were linearized by incubation for 30 min at 37°C either with restriction enzymes *Not*I and *Eco*RI (pHC, NEB) or with *Not*I and *Bam*HI (pLC, NEB) at a concentration of 20 U/ml and 2 µg pDest per 100 µl reaction in CutSmart buffer (NEB). Electroporation into yeast was accomplished using 12 µg CDR-H3 PCR product (VH PCR products pooled at equimolar ratios) and 3.5 µg digested pHC destination plasmid per transformation reaction in EBY100 according to Benatuil et al. (30). Ten electroporation reactions were performed in parallel for CDR-H3 library construction. Library size was calculated from serial dilution plating of transformed cells on SD-Trp agar plates. The light chain plasmid was generated accordingly in BJ5464. To combine generated VH diversity with the fixed light chain, yeast mating was applied (31). To this end, haploid EBY100 cells harboring the heavy chain diversity and BJ5464 cells containing the single light chain were grown in SD media, lacking either tryptophan or leucine, for 24 h at 30°C. Afterwards, cells were pooled and incubated on YPD agar plated at 30°C for 24 h. Subsequently, cells were transferred into SD media lacking both amino acids. Library size was calculated by dilution plating on SD-Trp-Leu agar plates.

### YSD Library Sorting

For library sorting, cells were grown overnight in SD-Trp-Leu medium at 30°C and 120 rpm. Next day, cells were harvested by centrifugation and used to inoculate SG-Trp-Leu medium at an OD_600_ of 1.0 and incubated for 2 days at 20°C. Fab expression was detected by incubation with light chain specific goat F(ab′)2 antihuman lambda R-phycoerythrin (R-PE) (SouthernBiotech, diluted 1:20). Antigen staining was conducted utilizing Penta-His Alexa Fluor 647 conjugate antibody (Qiagen, diluted 1:20) for sorting round 1 or SureLight^®^ APC Anti-6X His tag^®^ antibody (Abcam, diluted 1:20) for sorting round 2. For this, cells were harvested and washed twice with PBS (Sigma Aldrich) prior to incubation with EGFR at a concentration of 1 µM for 30 min on ice. After washing thrice, cells were then incubated with secondary labeling reagents for another 30 min on ice. Finally, cells were washed thrice with PBS and resuspended in appropriate volume for FACS sorting on a BD FACSAria™ Fusion cell sorter (BD Biosciences).

### Antibody Expression and Purification

Constructs were designed in-house as knob-Fc (human IgG1) fusion proteins or chimeric IgGs comprising human constant regions (IgG1), synthesized and subcloned into pTT5 plasmid backbone at GeneArt (Thermo Fisher Scientific) for transient expression in Expi293™ cells. To this end, 25 ml Expi293™ cells were transiently transfected with expression vectors according to the manufacturer’s recommendations (Thermo Fisher Scientific). The antibody-containing supernatants were harvested 5 days posttransfection and purified *via* MabSelect antibody purification chromatography resin (GE Healthcare). For sample formulation in PBS, pH 6.8, a dialysis step using Pur-A-Lyzer™ Maxi 3500 Dialysis Kit (Sigma Aldrich/Merck KGaA) was performed for 24 h at 4°C with a subsequent protein concentration step using Amicon Ultra-4 Centrifugal Filters (EMD Millipore), if necessary. The protein concentrations were determined *via* UV–Vis spectrophotometric measurement using the QIAexpert system (Qiagen), while aggregate formation was analyzed by analytical SEC of 10 μg protein per sample, using a TSKgel SuperSW3000 column (4.6 × 300 mm, Tosoh Bioscience LLC) in an Agilent HPLC system with a flowrate of 0.35 ml/min. Furthermore, gel electrophoresis analyses were performed to assess correct molecule assembly. To this end, 5 µg of protein was loaded onto NuPage™ 4-12% Bis–Tris gels and separated for 30 min at 200 V. For the initial sample preparation, 25 µg of protein was mixed with NuPAGE^®^ LDS Sample Buffer (4×) (Invitrogen) and NuPAGE^®^ Sample Reducing Agent (10×) (Invitrogen), in case of reducing gels, in a total volume of 50 µl (PBS) and incubated at 70°C for 15 min. For non-reducing conditions, samples were treated with NuPAGE^®^ LDS Sample Buffer (4×) only and heated for 1 min at 70°C prior to separation. As protein ladder, SeeBlue™ Plus 2 Prestained Standard (Invitrogen) was used.

### Biolayer Interferometry

To assess binding capacities of the expressed proteins to recombinant antigen protein, an Octet RED96 system (ForteBio, Pall Life Science) was used with 1,000 rpm agitation at 25°C for all experiments. For initial binding confirmation of the bovine × human chimeric molecules, proteins were loaded at 5 µg/ml in PBS for 180 s on antihuman-FC (AHC) biosensors prior to a 60 s sensor rinsing step in kinetics buffer [KB; PBS, 0.1% (v/v) Tween-20 and 1% (w/v) BSA]. Association to recombinant human EGFR extracellular domain (produced in-house) was measured afterwards for 180 s using 100 nM of EGFR, followed by dissociation for 120 s in KB. For kinetics analysis, molecules were also loaded on AHC biosensors for 180 s at 5 μg/ml in PBS followed by a sensor rinsing step in KB for 60 s. Subsequently, association to rh EGFR was measured for 300 s in varying concentrations ranging from 3.125 to 200 nM in KB followed by dissociation in KB for 300 s. To scrutinize a possible epitope overlap of the expressed proteins and control molecules with either Cetuximab (produced in-house), Matuzumab (produced in-house), or natural ligand EGF (Sino Biological Inc.), competition analysis was performed by loading EGFR *via* its histidine-tag on anti-Penta His (HIS1K) biosensors at 5 μg/ml in PBS for 3 min. After a step of sensor rinsing in KB for 60 s, association to the expressed proteins was performed for 300 s at 100 nM followed either by dissociation in KB or by another association step with Cetuximab, Matuzumab, or EGF, all supplemented with the respective protein utilized for first association at 100 nM. Antibodies targeting the same EGFR subdomain were further analyzed for competition in both orientations between each other according to the competition experiments with EGF at association and dissociation times of 180 s, respectively. Moreover, for every biolayer interferometry (BLI) experiment, appropriate negative controls were included, e.g., an unloaded sensor control to analyze unspecific association of the antigen (K_D_ determination) or antibody (competition assay) and unrelated antigen to validate specificities. Resulting data was fitted and analyzed with ForteBio data analysis software 8.0 using a 1:1 binding model after Savitzky–Golay filtering.

### Flow Cytometry

To assess cellular binding properties of chimeric IgGs or Knobbodies, a Guava^®^ easyCyte 12HT device (Merck Millipore) with a guavaSoft 3.2 Software system was used. For each experiment, a total number of 5,000 EGFR-positive A431 or EGFR-negative ExpiCHO™ per well were measured (5,000 cells/well). To this end, 10^5^ cells/well were seeded and incubated for 1 h on ice with antibody derivatives in varying concentrations (0.05 nM to 1 µM) after two initial washing steps with PBS + 1 % (w/v) BSA. Following antibody incubation, two additional washing steps with PBS + 1% (w/v) BSA were performed with subsequent Alexa Fluor^®^ 488 AffiniPure Fab Fragment Goat Antihuman IgG (H + L) (Jackson ImmunoResearch) detection antibody staining (1.1 µM) at 4°C for another 30 min. After two washing steps with PBS + 1% (w/v) BSA, 20 μg/ml propidium iodide (Invitrogen) was used to label dead cells in a total volume of 200 μl/well. Controls were included, e.g., cells without antibody incubation and cells labeled with the detection reagent only. Moreover, Cetuximab was used as a control to determine EGFR expression on both cell lines. Binding of CD16a to the bovine-derived chimeric molecules was determined using a Sartorius iQue3 flow cytometer. For the assay, 10^5^ cells/well A431 cells were seeded and incubated for 40 min on ice with antibody samples at 200 nM. After centrifugation and removal of the supernatant, cells were incubated for 30 min with polyhistidine-tagged CD16a (R&D Systems) at 200 nM on ice, followed by additional 30 min incubation with the detection antibody at 200 nM [Ms mAb to 6× His tag (PE) (Abcam)]. Prior to the measurement, cells were spun down and resuspended in 100 µl BSA + 1% BSA containing 20 µg/ml propidium iodide (Invitrogen) for dead cell staining. Assay controls, e.g., untreated cells and cells treated without addition of detection antibody, antibody samples or CD16a, respectively, and cells treated with Cetuximab, Matuzumab, or anti-HEL IgG (produced inhouse) were used. For analysis, FlowJo™ 10.2 software was employed.

### Epitope Mapping on the Subdomain Level *via* YSD

YSD-based EGFR epitope mapping was performed using yeast cells displaying six different truncated versions of the extracellular potion of EGFR (amino acids 1–124, 1–176, 1–294, 273–621, 294–543, and 475–621, respectively), as described previously (32, 33). Cells were harvested by centrifugation, washed once with PBS-B [PBS + 0.1% (w/v) BSA] and incubated with 200 nM (500 nM for Cetuximab) of the respective antibody variant for 30 min on ice. After washing once with PBS-B, antibody binding was verified using a goat antihuman IgG-Fc-PE conjugate (Fisher Scientific, diluted 1:20). Following another washing step, cells were screened by flow cytometry using a CytoFlex S (Beckman Coulter).

### Killing Assay

To determine the capacities of NK-mediated ADCC induction by knob-Fc fusion proteins and chimeric IgG molecules, fluorescence-microscopy-based tumor cell killing assays were performed using an Incucyte^®^ Live Cell Analysis System (Sartorius). For this, peripheral blood mononuclear cell (PBMCs) were isolated from whole blood samples of healthy human donors by density gradient centrifugation prior to NK cell isolation *via* EasySep™ Human NK Cell Isolation Kit (Stemcell Technologies). After overnight incubation in AIM V medium (Gibco) supplemented with 100 U/ml recombinant IL-2 (R&D Systems), effector cells were adjusted to 0.625 × 10^6^ vc/ml. The EGFR-positive cell line A431 and EGFR-negative ExpiCHO™ cells were prepared in parallel by staining with CellTracker™ Deep Red Dye (Thermo Fisher) according to the manufacturer’s instructions. Stained target cells were seeded in a 384-well clear bottom microtiter plate (Greiner Bio-One) at 2,500 cells/well in 20 µl volume and incubated for 3 h. Subsequently, 20 µl NK cell suspension was added, resulting in an effector cell to target cell (E:T) ratio of 5:1. Afterwards, 5 µl antibody solution with varying concentrations ranging from 0.005 pM to 500 nM and SYTOX™ Green Dead Cell Stain (Invitrogen, 0.03 µM) were dispensed to the assay followed by plate incubation and online measurement for 24 h in the Incucyte^®^ system. Parallel incubation of control wells containing target cells cultivated with NK cells in the absence of antibodies, target cells only, and target cells cultivated with 30 µM staurosporine (Merck Millipore) for maximal tumor cell lysis, allowed for the identification of dead target cells only by fluorescence overlay (green and red fluorescence) analysis. To allow for a thorough comparison, maximal killing capacities were normalized to Cetuximab.

### AKT Pathway Signaling Assay

To investigate AKT signaling, either the Phospho-AKT1/2/3 (Ser473) HTRF Kit (64AKSPEG, Cisbio/Perkin Elmer) or the Akt Signaling Whole Cell Lysate Kit (Mesoscale Discovery) were employed. In case of the phospho-AKT1/2/3 kit (64AKSPEG, Cisbio/Perkin Elmer), A549 cells were seeded onto sterile cell culture 96-well plates at a final cell density of 3.5 × 10^4^ cells/well in complete growth medium. After adherence, cells were serum starved in Dulbecco’s modified Eagle’s medium (DMEM) overnight. The following day, cells were pretreated with the respective antibodies in a concentration range from 2.6 µM to 0.62 pM in a fourfold dilution series and incubated for 1 h in serum-free medium. Then, EGF (20 ng/ml) was added to the cells and incubated for 10 min at 37°C. Subsequently, the cells were lysed using supplemented lysis buffer from the phospho-AKT1/2/3 (Ser473) HTRF Kit (64AKSPEG, Cisbio/Perkin Elmer). After 30 min incubation, the lysates were analyzed following the manufacturer’s protocol. Dose–response curves were fitted with a non-linear regression using GraphPad Prism 8.0.1. Biological duplicates were measured for each variant. For the Akt Signaling Whole Cell Lysate Kit, A549 cells were seeded into sterile cell culture 48-well plates at a final cell density of 1 × 10^5^ cells/well in complete growth medium. After adherence, cells were serum starved in DMEM overnight. For antibody treatment, cells were pretreated with the respective antibody at 667 nM for 1 h in serum-free medium. Then, EGF solution (20 ng/ml) was added to the cells and incubated for 10 min at 37°C. Subsequently, the cells were rinsed once with ice-cold PBS and lysed using Complete Lysis Buffer from Akt Signaling Whole Cell Lysate Kit (MesoScale Discovery). For phosphoprotein analysis, the lysates were measured following the manufacturer’s protocol. One-way ANOVA comparison was performed to statistically analyze the results considering values of p ≤0.05 as significant.

## Results

### Isolation of Chimeric EGFR-Specific Ultralong CDR-H3 Fab Fragments

**Figure 2:**
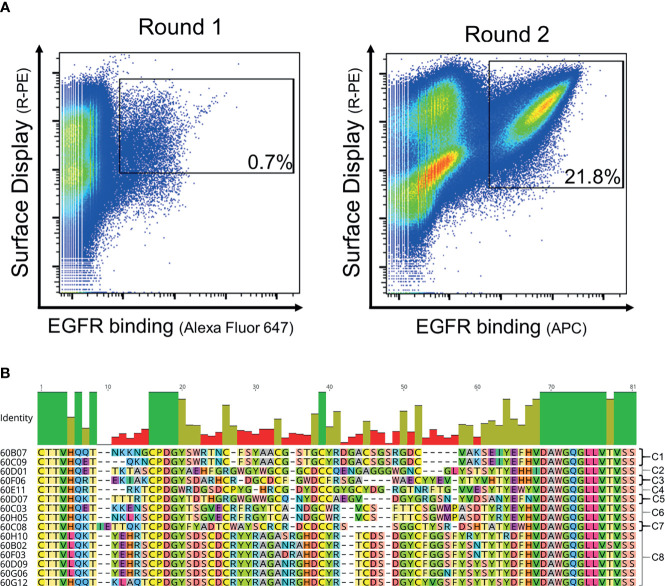
FACS-based selection for the enrichment of EGFR-specific chimeric bovine × human Fab fragments by yeast surface display and sequence analysis of the sorting output. **(A)** Two rounds of sorting were performed to isolate EGFR-specific antibodies. A two-dimensional sorting strategy was implemented to simultaneously label for functional Fab display and EGFR binding. To this end, library cells were incubated with recombinant human his-tagged EGFR at a concentration of 1 µM followed by staining using secondary detection reagents directed against the his-tag and against the constant region of the human lambda chain. **(B)** CDR-H3 sequence alignment of unique ultralong chimeric antibody clones obtained from library sorting. Sequence of IGHJ2-4 is included and clone names. Based on sequence similarity, eight clonotypes were identified as indicated. Green bars highlight 100% sequence identity at a particular residue, olive bars indicate an intermediate sequence conservation, and red bars represent a high sequence diversity. Amino acids given in one-letter code and in different colors. Alignment generated with Geneious Prime^®^ v2021.1.1.

In order to generate EGFR-specific ultralong CDR-H3 antibodies by yeast surface display following cattle immunization, we developed a strategy that relies on specific ultralong CDR-H3 amplification and subsequent engraftment of this region onto bovine/human chimeric Fab fragments (**Figure 1A**). Immunization resulted in a detectable total IgG titer for all animals utilized with two animals showing a more robust response (**Supplementary Figure S1**). For PCR-based amplification, primers specifically targeting FR3 of IGHV1-7 and FR4 were designed (**Supplementary Table S1**). There is a strong bias for ultralong CDR-H3 antibodies utilizing the IGHV1-7 segment in cattle. In fact, more than 90% of bovine ultralong CDR-H3 antibodies belong to IGHV1–7 (14). This gene segment comprises a nucleotide extension at its 3′ end (12), resulting in a longer V-region. Oligonucleotide mixtures were designed that specifically target this nucleotide duplication. A pYD-derived heavy chain display plasmid (pHC) was constructed harboring a synthetic bovine IGHV1–7 domain fused to human constant region CH1 and AGA2P (**Supplementary Figure S2A**). However, CDR-H3 was replaced by a stuffer sequence (comprising multiple stop codons in every reading frame and *Not*I and *EcoR*I cleavage sites for vector linearization). After immunization, RNA extraction from PBMCs, and cDNA synthesis and ultralong CDR-H3 region amplification, the heavy chain library was constructed by engraftment into linearized pHC in a homologous recombination-based process referred to as plasmid gap repair employing yeast strain EBY100 (haploid, mating type a, MATa). This procedure yielded a calculated library size of approximately 5 × 10^7^ independent clones. Since it is believed that the major function of the light chain is in stabilizing the paratope (12), we decided to display this diversity with a single light chain (V30) that was deduced from pdb entries: 4K3E (21), 5E99, 5IHU, 5IJV, 5ILT (13), 6E9G, 6E9H, 6E9I, 6E9K, 6E9Q, and 6E9U (15), which share a CDR-identical light chain. This variable domain (**Supplementary Figure S2B**) was incorporated into a linearized light chain plasmid (pLC) by gap repair cloning and consequently fused to human CLλ exploiting BJ5464 cells (haploid, mating type α, MATα). Subsequently, yeast mating was applied to establish the chimeric Fab library (29), resulting in adequate oversampling of the estimated heavy chain diversity (about 3 × 10^9^ unique clones). To assess the functionality and quality of the generated library, 96 clones were sent out for sequencing. This revealed a high functionality of ultralong CDR-H3 sequences of 79.2% (76/96 single clones), with 3.1% (3/96 clones) sequences having a CDR-H3 region of <40 amino acids and 12.5% (12/96 sequences) frame-shifted sequences and 5.2% ambiguous results (5/96). Afterwards, this library was subjected to fluorescence-activated cell sorting (FACS) using a target concentration of 1 µM rh EGFR (ECD). As shown in **Figure 2A**, within two rounds of selection, we were able to enrich for an antigen-binding population. Sequencing revealed 15 unique ultralong CDR-H3 regions ranging from 59 to 65 residues (**Figure 2B**). Based on sequence similarity of the knob region, those were clustered into eight clonotypes. Interestingly, the CDRs only harbored an even number of four to eight Cys residues, presumably forming disulfide bonds, a hallmark of ultralong CDR-H3-derived knob structures.

### Biophysical and Biochemical Characterization of Chimeric Ultralong CDR-H3 IgG Antibodies

Identified unique paratopes were reformatted and produced as chimeric IgG1 antibody derivatives utilizing human constant regions (**Figure 1B**). In an initial binding assay employing biolayer interferometry (BLI) at an antigen concentration of 100 nM, five out of eight groups of different clonotypes comprised variants that were EGFR specific with six different unique variants in total (**Supplementary Figure S3**). Consequently, only EGFR-specific ultralong CDR-H3 entities were scrutinized more meticulously. From a biophysical perspective, expression yields of all EGFR-targeting chimeric IgG-like molecules post protein A purification were in the triple digit milligram per liter scale (**Table 1**). Given the additional disulfide bond patterns resulting in a higher complexity of these kind of molecules compared to standard IgGs (e.g., obtained from rodents), the expression yields of chimeric ultralong CDR-H3 antibodies can be considered as rather favorable (**Table 1**). In addition, size exclusion chromatography (SEC) profiles revealed more than 85% target monomer peaks for all clones, with 60C03 IgG, 60C08 IgG, 60F06 IgG, and 60H05 IgG displaying target peaks of more than 95% (**Table 1** and **Supplementary Figure S4**). Importantly, main target peaks were in the same range as for therapeutic antibodies Cetuximab and Matuzumab. Since cattle-derived molecules have not been optimized for developability within this study, this is generally indicating beneficial biophysical properties of herein generated ultralong CDR-H3 entities (**Table 1**). Of note, 60D01 IgG eluted later than expected (**Supplementary Figure S4**). Since sodium dodecyl sulfate–polyacrylamide gel electrophoresis (SDS-PAGE) under non-reducing and reducing conditions showed main bands at the expected sizes (**Supplementary Figure S5**), this can be attributed to interaction of the molecule with the column matrix, and consequently, the peak at approximately 10.5 min was considered as target species. Essentially, SDS-PAGE analysis revealed main bands with the expected molecular weight and high purities for all chimeric IgGs similar to Cetuximab and Matuzumab (**Supplementary Figure S5**).

**Table 1:** Characterization of chimeric bovine × human ultralong CDR-H3 antibodies.

Molecule	Yield (mg/L)	SEC (%)	KD (M)	k on (1/Ms)	k off (1/s)	Comp. EGF	Comp. Cetuximab	Comp. Matuzumab	Domain targeting	Epitope bin	EC_50_ cell binding (nM)	ADCC EC_50_ killing (pM)	ADCC Mean max killing (normalized to Cetuximab) (%)
60C03 IgG	272	98.3	1.371E−08	1.43E+05	1.96E−03	no	no	no	II	3	3.0	26.5	103.1
60C08 IgG	168	95.3	4.778E−08	5.97E+05	2.85E−02	no	no	no	I–II	1	0.9	58.7	92.1
60D01 IgG	145	86.1	1.20E−07	6.24E+05	7.49E−02	no	no	no	IV	5	1.3	605.9	76.3
60E11 IgG	182	88.6	4.103E−09	5.42E+05	2.22E−03	no	no	no	I–II	2	10.1	3.3	96.0
60F06 IgG	151	98.8	7.385E−09	1.18E+05	8.70E−04	no	no	no	II	4	3.0	4.8	107.5
60H05 IgG	193	98.1	2.79E−08	1.14E+05	3.19E−03	no	no	no	II	3	2.6	16.4	104.6

Expression yields were determined after protein A purification. Analytical SEC was utilized to determine the percentage of target monomer species. Affinities and competition assays were performed via BLI. Domain targeting was analyzed by cell binding assays using yeast surface-displayed fragments of the extracellular portion of EGFR. For cell binding studies and ADCC assays EGFR-overexpressing A431 cells were employed.

**Figure 3:**
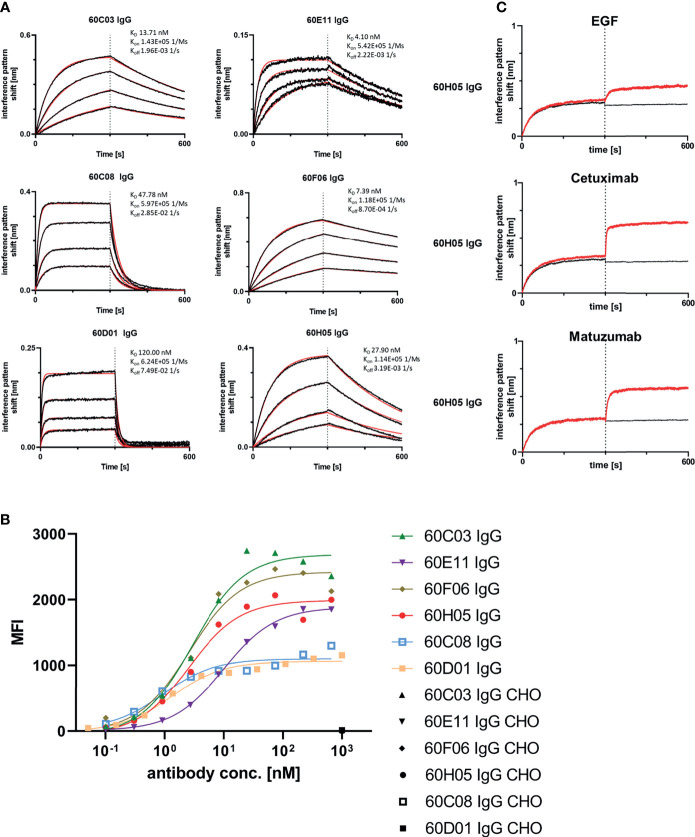
Biochemical characterization of chimeric ultralong CDR-H3 IgG antibodies. **(A)** Kinetic measurements against recombinant human EGFR extracellular portion. IgGs were loaded onto sensor tips. After sensor rinsing, antigen binding was conducted at different concentrations (100, 50, 25, and 12.5 nM for all molecules except for 60D01 IgG. Here, concentrations were 200, 100, 50, and 25 nM) for 300 s, followed by a dissociation step in kinetics buffer for 300 s. **(B)** Cellular binding of chimeric ultralong CDR-H3 molecules on EGFR-positive cell line A431 and EGFR-negative ExpiCHO™ cells (only shown at a concentration of 1 µM). **(C)** Non-competitive binding of 60H05 IgG (as representative clone) to recombinant human EGFR extracellular region with EGF-Fc fusion, Cetuximab, and Matuzumab as determined *via* BLI. EGFR was loaded onto sensor tips. In a first association step, chimeric IgGs were utilized at a concentration of 100 nM. In a second association step, EGF-Fc fusion, Cetuximab, or Matuzumab was used at 100 nM and first analyte at 100 nM.

Next, we aimed at characterizing the biochemical properties of the herein generated molecules. To this end, BLI measurements were exploited to analyze binding kinetics (**Figure 3A**; **Table 1**; **Supplementary Table S2**). Affinities ranged from single digit nanomolar EGFR binding for 60E11 IgG and 60F06 IgG to targeting in the lower triple digit nanomolar range 60D01 IgG (K_D_ = 120.0 nM). Intriguingly, this high-affinity binding behavior of 60E11 (K_D_ = 4.1 nM) and 60F06 (K_D_ = 7.4 nM) resulted from initial engraftments of CDR-H3 regions only onto a fixed, synthetic Fab backbone, clearly corroborating the paramount importance of this region for binding in bovine ultralong antibodies. Importantly, affinities for most of the antibodies were in the same range as Cetuximab (K_D_ = 1.9 nM) and Matuzumab (K_D_ = 16.6 nM) (**Supplementary Figure 6**; **Supplementary Table 2**). Additionally, we performed cellular binding assays using EGFR-overexpressing tumor cell line A431 and EGFR-negative ExpiCHO™ cells (**Figure 3B**). All ultralong CDR-H3 IgG antibody variants showed specific binding to EGFR-overexpressing cells with apparent binding affinities in the lower nanomolar range (EC_50_ cell binding ranging from 0.9 to 10.1 nM, **Table 1**), whereas no significant binding was observed for CHO cells, clearly demonstrating highly specific binding capacities of isolated variants.

Competition assays by BLI of all six ultralong CDR-H3 antibodies revealed non-competitive binding with recombinant human EGF to EGFR as exemplarily shown for 60H05 IgG in **Figure 3C** (and **Supplementary Figure S7**). This is in contrast to therapeutic antibody Cetuximab that competed with recombinant human EGF for binding to EGFR, whereas Matuzumab demonstrated non-competitive binding (**Supplementary Figure S8**). Accordingly, none of the generated chimeric antibodies showed competition with Cetuximab (**Figure 3C**; **Supplementary Figure S7**). Interestingly, all cattle-derived antibodies also did not compete with Matuzumab for binding to EGFR (**Figure 3C**, **Supplementary Figure S7**). To get a more comprehensive picture about epitope targeting to the four different extracellular subdomains of EGFR, we employed flow cytometric analysis using yeast cells displaying truncated fragments of the EGFR extracellular region, as described previously (32, 33) (**Supplementary Figures S9**, **S10**). To this end, yeast cells were incubated with the respective cattle-derived chimeric antibody followed by subsequent staining using a goat antihuman Fc antibody PE conjugate. As opposed to Cetuximab that shares a similar main epitope on EGFR as EGF and for which we were able to map binding to subdomain III on EGFR, none of the generated antibodies bound to yeast-displayed fragments corresponding to this particular domain. We were able to map binding of 60C03 and 60H05 to domain II of EGFR, while 60D01 targeted subdomain IV (**Table 1**). Interestingly, clones 60C08 and 60E11 showed binding to displayed fragments of EGFR corresponding to the interface region of domains I and II, whereas no binding was observed for those surface-expressed fragments that represent regions of these respective domains, which do not contain this switch region. These findings corroborate the notion that 60C08 and 60E11 target the transition region between EGFR subdomains I and II. Besides, clone 60F06 exhibited only moderate binding to yeast cells displaying an EGFR fragment comprising amino acid residues 1–294, whereas no binding was detected to all other surface-expressed fragments of EGFR. Hence, it is tempting to speculate that this ultralong CDR-H3 antibody binds to an epitope of domain II of EGFR that is not presented appropriately on the surface of *S. cerevisiae*. To enable a more precise epitope discrimination of chimeric IgGs targeting the same subdomain on EGFR, we performed BLI-based competition assays using 60C08 IgG and 60E11 IgG (targeting the transition region of EGFR domain I and II) and 60C03 IgG, 60F06 IgG, and 60H05 IgG (mapped to domain II, **Supplementary Figure S11**). Interestingly, when 60C08 IgG was exploited for the first association and 60E11 for the second step, we observed complete competition, whereas *vice versa*, partial competition, i.e., a diminished second association was seen. This suggests that the epitopes of both antibodies are overlapping but not identical. For antibodies that were mapped to subdomain II on EGFR, we saw competition for 60C03 and 60H05 IgGs. This was expected since both antibodies share a very similar paratope (**Figure 2B**). However, 60F06 did not compete with any of those domain II-specific antibodies and thus addresses a different epitope. Ultimately, albeit none of the ultralong CDR-H3 antibodies competed for binding with EGF, Cetuximab, or Matuzumab, respectively, a rather broad epitope coverage with five epitope bins was observed.

### Functional Characterization of Chimeric Ultralong CDR-H3 Antibodies

**Figure 4:**
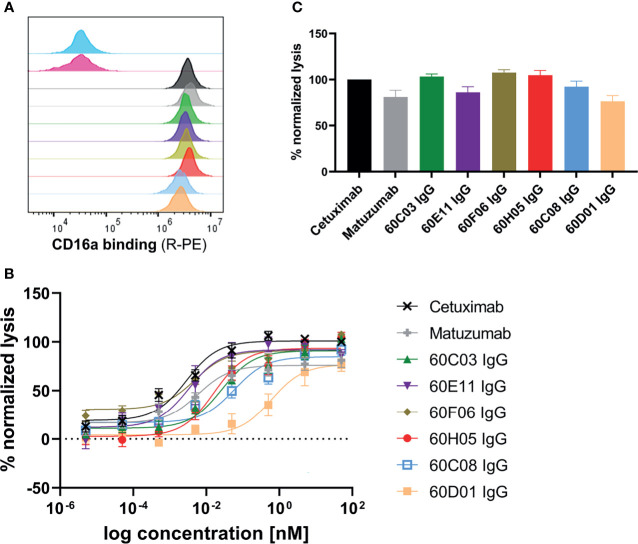
ADCC capacities of generated chimeric ultralong CDR-H3 antibodies. **(A)** Simultaneous binding to EGFR-overexpressing cell line A431 and recombinant human FcγRIIIa as determined by flow cytometry. A431 cells were labeled with respective antibody entity at 200 nM followed by incubation with 200 nM his-tagged FcγRIIIa protein and 200 nM detection antibody. Controls were included, i.e., all detection reagents including FcγRIIIa without primary antibody (cyan), unrelated antibody (anti-HEL IgG1, pink), Cetuximab (black), and Matuzumab (gray). Bovine-derived chimeric IgGs shown in different colors: 60C03 IgG (green), 60E11 IgG (purple), 60F06 IgG (gold), 60H05 IgG (red), 60C08 IgG (light blue), and 60D01 IgG (orange). **(B, C)** Fluorescence-microscopy-based killing assay using EGFR-positive A431 target cells and PBMC-purified NK effector cells at an E:T ratio of 5:1. Analysis of dose-dependent **(B)** and maximum **(C)** target cell killing *via* NK cell-mediated ADCC. Cetuximab (black) and Matuzumab (gray) were included. Data were normalized to allow comparison of the independent experiments. Graphs show normalized means ± SEM of n = 8 (except for 60D01 IgG n = 4) different healthy donors.

One effector mechanism mediated by IgG isotypes considered as important for many therapeutic anticancer antibodies is referred to as antibody-dependent cell-mediated cytotoxicity (ADCC) (34). This function relies on bridging the low-affinity activating FcγRIIIa (CD16a) that is expressed on the majority of NK cells with cells opsonized with IgG antibodies, resulting in potent NK cell degranulation and target cell lysis. In order to investigate whether the generated chimeric ultralong CDR-H3 IgG antibodies induce ADCC, we first analyzed simultaneous binding to EGFR-overexpressing cell line A431 and FcγRIIIa using an indirect FACS-based assay. Herein, A431 cells were incubated with the respective antibody derivative, followed by recombinant his-tagged FcγRIIIa incubation and detection *via* an anti-his tag antibody fluorophore conjugate. Thereby, we were able to detect specific binding of all generated cattle-derived antibodies to EGFR and FcγRIIIa similar to Cetuximab and Matuzumab (**Figure 4A**). In accordance with slightly diminished maximal binding capacities of 60C08 IgG and 60D01 IgG on A431 and lower affinities measured with recombinant EGFR, fluorescence intensities were also slightly reduced for both variants. Subsequently, all variants were tested for their capacities to elicit tumor cell killing using EGFR-overexpressing tumor cell line A431 and PBMCs-isolated NK cells as an effector cell population. To enable a thorough comparison to therapeutic EGFR-targeting antibodies either approved for therapy or investigated in clinical trials, we included antibodies Cetuximab as well as Matuzumab and normalized maximal killing capacities to Cetuximab (**Figures 4B, C**). Both therapeutic antibodies elicited NK cell mediated tumor cell lysis with potencies (EC_50_ killing) in the single digit picomolar range (EC_50_killing = 2.8 pM for Cetuximab and EC_50_killing = 6.0 pM for Matuzumab). Maximal killing capacities, however, were diminished for Matuzumab compared to Cetuximab (81% in direct comparison at a concentration of 50 nM). All six cattle-derived chimeric ultralong CDR-H3 IgGs exerted significant NK cell-mediated killing of A431 cells, whereas neglectable lysis was observed for EGFR-negative ExpiCHO™ cells at substantially higher concentrations (**Figure 4** and **Supplementary Figure S12**). Potencies varied from the single digit picomolar range to the triple digit picomolar range with clones 60E11 (EC_50_killing = 3.3 pM) as well as 60F06 (EC_50_killing = 4.8 pM) demonstrating similar ADCC activities as therapeutic antibody Cetuximab (**Table 1**). In general, efficacies ranged from 76% maximal normalized lysis of A431 (clone 60D01) to 108% maximum killing compared to Cetuximab (clone 60F06) (**Table 1**). We also set out to analyze the kinetic of tumor cell killing mediated by ultralong CDR-H3 antibodies. For this, an antibody concentration of 5 pM was chosen, since this roughly corresponds to the potencies of Cetuximab, Matuzumab, 60E11 IgG, and 60F06 IgG. As shown in **Supplementary Figure S13**, time-resolved lysis capacities of most of the variants were comparable to Cetuximab or Matuzumab, with most of NK cell-mediated killing occurring within the first 10 h. Subtle differences were observed for the first 8 h for instance for 60E11 IgG and for 60F06 IgG that display similar potencies. While for 60F06, we were already able to detect killing similar to Matuzumab at 4 h, 60E11 IgG seemed to elicit slightly lower levels of killing at this time point. Considerably, maximal lysis capacities for the constructs differed enormously among the tested compounds. This can be attributed to differences within EC_50_killing for the different antibodies. In this respect, potencies of 60D01 IgG were in the triple digit picomolar range. Hence, barely any killing was detected for this variant at 5 pM. Taken together, cattle-derived ultralong CDR-H3 antibodies are capable of mediating potent and robust target-dependent ADCC when grafted onto a human IgG1 backbone.

**Figure 5:**
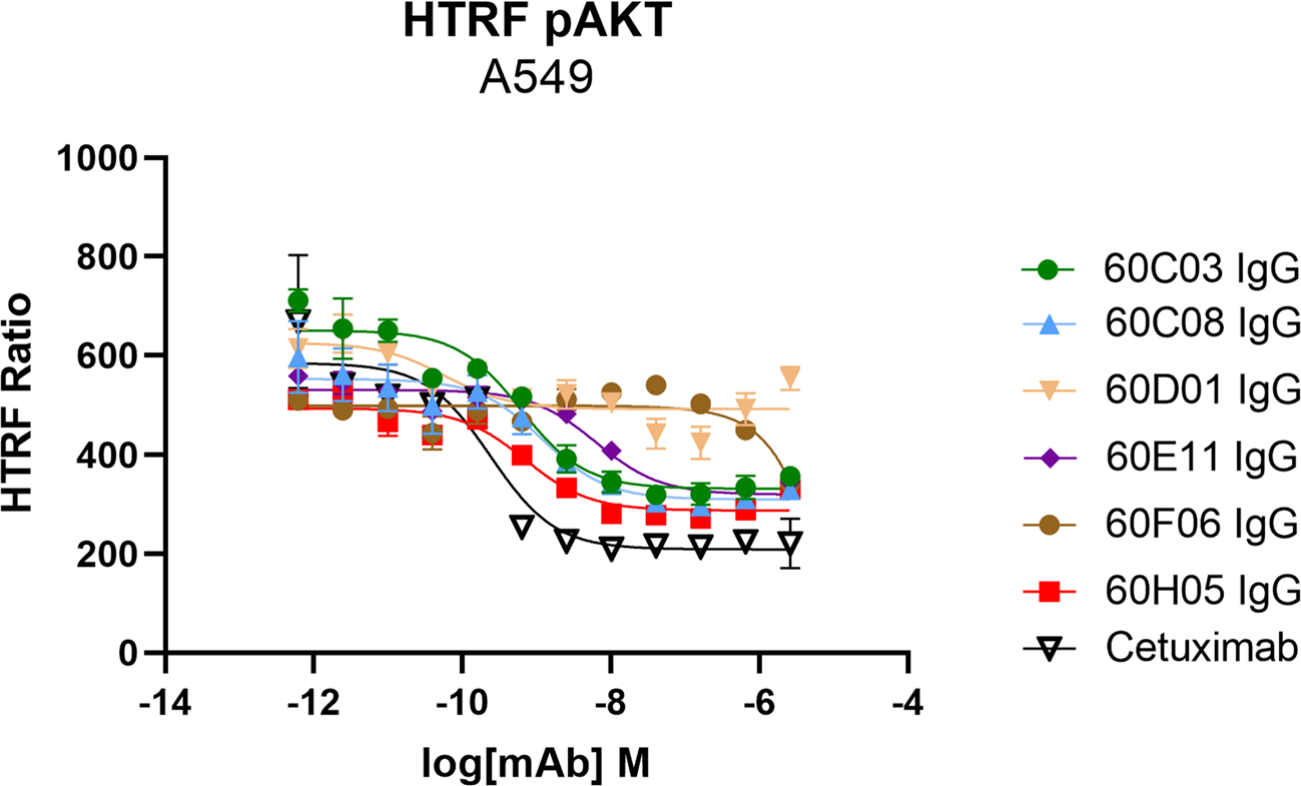
Inhibition of EGFR-dependent AKT phosphorylation of chimeric ultralong CDR-H3 antibodies. EGFR-positive A549 cells were incubated with the respective antibody derivatives in different concentrations starting from 2.6 µM to 0.62 pM in a fourfold dilution series. Subsequently, EGFR-mediated downstream signaling (at a concentration of 20 ng/ml) was monitored by quantitative determination of phospho-AKT. All samples were measured in duplicates. Homogeneous time-resolved fluorescence ratio (HTRF ratio) was calculated according to the manufacturer’s instructions as follows: acceptor (665 nm)/donor (620 nm) × 10,000.

Another important mode of action of therapeutic antibodies against receptor tyrosine kinases is the inhibition of receptor-mediated downstream signaling. In this respect, Cetuximab binds to the extracellular region of EGFR, blocking the interaction of EGFR with EGF, the natural ligand. Therefore, Cetuximab blocks the signal transduction pathway providing several antitumor effects such as cell-cycle arrest or induction of apoptosis (28, 35). In order to see whether the generated ultralong CDR-H3 antibodies can inhibit signal transduction, we performed AKT pathway signaling assays, which are triggered by EGFR. To this end, phosphorylation of AKT by EGF stimulation of EGFR-overexpressing A549 cells was analyzed in the presence or absence of chimeric antibodies at different concentrations (**Figure 5**). Besides 60D01 IgG showing no inhibition and 60F06 IgG eliciting only partial inhibition at high concentrations, all ultralong CDR-H3 antibodies significantly inhibited AKT phosphorylation in a concentration-dependent manner. Half maximal inhibition ranged from the triple digit picomolar range for 60C03 IgG (IC_50_ = 615 pM) and 60H05 IgG (IC_50_ = 695 pM) to single digit nanomolar inhibition for 60C08 IgG (IC_50_ = 1.2 nM) and 60E11 IgG (IC_50_ = 6.4 nM), whereas Cetuximab impeded most potently AKT signaling (IC_50_ = 241 pM).

### Generation and Characterization of Knob-Fc Fusion Proteins (Knobbodies)

We also set out to examine whether the disulfide-rich knob regions derived from YSD were capable of independently functioning as a paratope without the antibody scaffold and without the stalk region. To this end, the knob regions of all six antigen-specific ultralong CDR-H3 antibodies (**Figure 1C** and **Supplementary Figure S3**) were grafted onto the hinge region of human IgG1 and expressed as Fc fusion proteins. Most clones displayed a tendency towards high aggregation as indicated by SEC target monomer peaks after protein A purification and SDS-PAGE analysis (**Table 2** and **Supplementary Figures S4**, **S5**). Intriguingly, clones 60C03 and 60H05 showed acceptable biophysical properties as indicated by SEC target monomer peaks of 87% and 80%, respectively (**Supplementary Figure S4**), and expression yields in the higher double digit milligram per liter scale. Consequently, only these clones were considered for further characterization. Interestingly, from a sequence perspective, both clones only differ in the switch region between the stalk and the knob with three amino acids difference in total (**Supplementary Figure S14**).

**Table 2:** Characterization of knob-human Fc (IgG1) fusion proteins (Knobbodies).

Molecule	Yield (mg/L)	SEC (%)	KD (M)	k on (1/Ms)	k off (1/s)	Comp. EGF	Comp. Cetuximab	Comp. Matuzumab	Domain targeting	EC_50_ cell binding (nM)	ADCC EC_50_ killing (pM)	ADCC Mean max killing (normalized to Cetuximab) (%)
60C03 Knobbody	76	87.0	3.661E−08	6.10E+04	2.23E−03	no	no	no	II	19.2	65.3	52.9
60H05 Knobbody	95	80.1	3.537E−08	5.35E+04	1.89E−03	no	no	no	II	15.2	62.1	58.1

Expression yields were determined post protein A chromatography. Target monomer peaks were determined by analytical SEC. Affinities and competition assays were performed via BLI. Domain targeting was analyzed by cell binding assays using yeast surface-displayed fragments of the extracellular region of EGFR. For cell binding studies and ADCC assays EGFR-overexpressing tumor cell line A431 was exploited.

**Figure 6:**
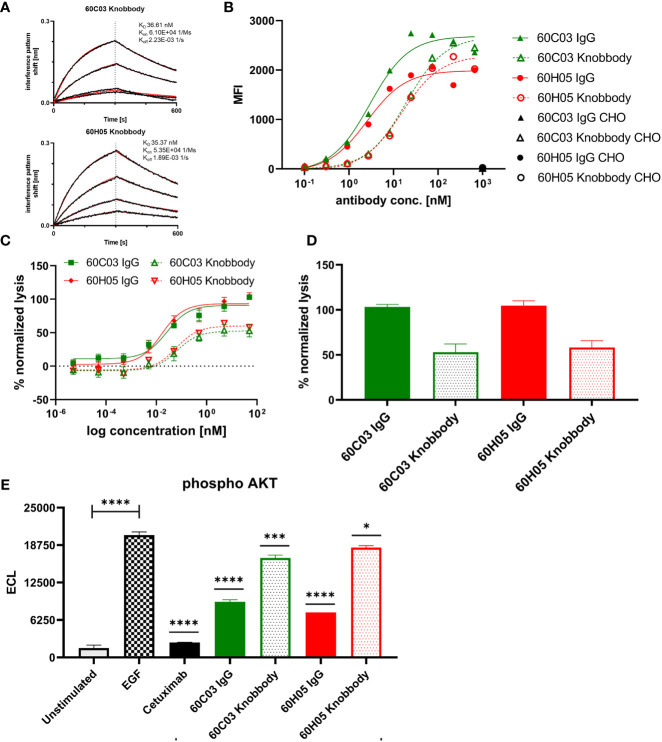
Biochemical and functional characterization of Knobbodies 60C03 and 60H05. **(A)** Kinetic measurements of Knobbodies 60C03 and 60H05 against recombinant human EGFR. Knobbodies were loaded onto sensor tips. After sensor rinsing, antigen binding was conducted at different concentrations (100, 50, 25, and 12.5 nM) for 300 s, followed by a dissociation step in kinetics buffer for 300 s. **(B)** Cellular binding of Knobbodies (dotted lines) in direct comparison to parental chimeric IgGs on EGFR-positive cell line A431 and EGFR-negative ExpiCHO™ cells (only shown at a concentration of 1 µM). **(C, D)** Cytotoxic activity of generated Knobbodies in direct comparison to their parental chimeric IgGs. Fluorescence-microscopy based killing assay using EGFR-positive A431 target cells and purified NK effector cells at an E:T ratio of 5:1. Analysis of dose-dependent **(C)** and maximum **(D)** target cell killing *via* NK cell-mediated ADCC. A comparison of 60C03 IgG (green) and 60H05 IgG (red) with their corresponding Knobbodies 60C03 (green, dotted) and 60H05 (red, dotted). Data were normalized to allow comparison of the independent experiments. Graphs show normalized means ± SEM of n = 8 different healthy donors. **(E)** Inhibition of EGFR-dependent AKT phosphorylation by Knobbodies 60C03 and 60H05. EGFR-positive A549 cells were incubated with respective Knobbodies and IgG counterparts at a concentration of 667 nM. Subsequently, EGF-mediated downstream signaling (at a concentration of 20 ng/ml) was monitored by quantitative determination of phosphor-AKT. Samples were measured in duplicates. ECL, electrochemiluminescence. ****p ≤ 0.0001, ***p ≤ 0.001, *p ≤ 0.05.

Affinities against recombinant human EGFR for both knob-Fc fusion proteins as determined by BLI were in the double digit nanomolar range (K_D_ = 36.6 nM for 60C03 Knobbody and K_D_ = 35.4 nM for 60H05 Knobbody) (**Figure 6A** and **Table 2**; **Supplementary Table S2**). In direct comparison with their parental IgG molecules, this represents a slight decline of approximately 2.7- and 1.3-fold, respectively (**Table 1**). Cellular binding assays on EGFR-overexpressing A431 cells and EGFR-negative ExpiCHO™ cells (**Figure 6B** and **Table 2**) revealed specific binding with apparent binding affinities in the double digit nanomolar range (EC_50_ cell binding = 19.2 nM for 60C03 Knobbody and EC_50_ cell binding = 15.2 nM for 60H05 Knobbody). In accordance with slightly diminished binding affinities using recombinant human EGFR, this means a drop in binding capacities of 6.4- and 5.8-fold, respectively (EC_50_ cell binding = 3.0 nM for 60C03 IgG and EC_50_ cell binding = 2.6 nM for 60H05 IgG). Equally to their parental IgG, none of both knob-Fc fusions competed with recombinant human EGF, Cetuximab or Matuzumab for binding to recombinant human EGFR (**Supplementary Figure S7**). Likewise, we were also able to map the epitope of both Knobbodies to subdomain II of EGFR (**Supplementary Figure S9** and **Table 2**).

We also exploited ADCC assays using EGFR-overexpressing cell line A431 and PBMC-isolated NK cells to scrutinize the generated Knobbodies in terms of biological functionality (**Figure 6C**). Both Knobbodies induced significant NK cell-mediated lysis of EGFR-positive A431 cells with negligible killing of EGFR-negative ExpiCHO™ cells (**Supplementary Figure S12**). 60C03 Knobbody and 60H05 Knobbody elicited half-maximal killing at concentrations of 65.3 and 62.1 pM, respectively (**Table 2**), representing a decrease in potencies of 2.5- and 3.8-fold compared to their parental IgGs (**Table 1**). Likewise, we also observed a loss of efficacies (maximum killing) for both moieties (**Figure 6D**). In this respect, maximal normalized lysis of A431 cells compared to Cetuximab were 52.9% for 60C03 Knobbody *versus* 103.1% for 60C03 IgG and 58.1% for 60H05 Knobbody *versus* 104.6% for the parental IgG counterpart.

Besides, both Knobbodies significantly inhibited downstream signaling of EGFR, as analyzed by AKT phosphorylation assays (**Figure 6E** and **Supplementary Figure S15**). However, compared to their parental antibody molecules, inhibition at a concentration of 667 nM was strongly diminished. These findings are in line with the reduced binding capacities of Knobbodies as opposed to their parental molecules, indicating that the antibody scaffold might be important for an optimal arrangement in terms of distance and orientation of the paratope that is mainly composed of the disulfide-rich knob architecture. Notwithstanding, these results are giving clear evidence that a subset of knob structures can function autonomously, without the ultimate need for stabilization and positioning as mediated by the stalk of the bovine ultralong CDR-H3 antibody backbone.

## Discussion

To efficiently generate antigen-specific bovine ultralong CDR-H3 paratopes, a platform process was established that relies on engrafting amplified CDR-H3 regions following immunization onto a single bovine × human chimeric Fab scaffold. Yeast surface display was employed to sample the resulting diversities. In this respect, yeast surface display has proven to be versatile for engineering different antibody derivatives (36–41) but also more complex molecules such as cytokines (42), ligands (43), or cysteine-rich miniproteins (44, 45). More recently, Sahin and colleagues could demonstrate that disulfide-rich peptides can also be enriched by phage display (46), substantiating the versatility of display technologies for engineering of more complex molecules. After immunization, an immune response was detected in all immunized cattle. However, since the total IgG titer was detected, it cannot be determined specifically how the response evolved regarding ultralong CDR-H3 antibodies and whether titers involved ultralong CDR-H3 entities in all specimen. For YSD library construction, oligonucleotides were designed that address the unique genetics of ultralong CDR-H3 antibodies. IGHV1-7 that is preferentially used for the formation of ultralong cow antibodies (14) comprises an eight nucleotide duplication in its 3′end (encoding for TTVHQ instead of AR/K) (12). This nucleotide extension has been specifically targeted by primer design, and sequencing of the initial library revealed a high functionality of clones displaying an ultralong CDR-H3 region (79.2%). Within two rounds of FACS-based selections, we were able to readily enrich for an antigen-binding population against EGFR that represents a promising target structure for anticancer therapy (26, 27). From sequencing the sorting output, we found more than a dozen CDR-H3 unique clones belonging to eight distinct clonotypes. In terms of diversity of ultralong CDR-H3 antibodies from cattle, this is comparable to what Burton and colleagues described for the isolation of HIV-targeting ultralong CDR-H3 antibodies (23). Moreover, this is also similar to the clonotypic output of antibodies targeting EGFR from other species such as camelids or chickens (29, 47). Binding assays revealed the successful isolation and generation of a panel of sequence-unique antibody derivatives with variants showing high-affinity binding in the single digit nanomolar range. This is remarkable, since all the sequence diversity of the library is harbored in region CDR-H3, whereas all other CDRs remain invariant. As already described by Smider and co-workers (13, 21), this is clearly corroborating the paramount role of CDR-H3 of ultralong bovine antibodies for antigen binding. In conclusion, YSD seems to be a valid platform technology for the isolation of ultralong CDR-H3 antibodies from cattle following immunization, particularly combined with the strategy to specifically amplify ultralong CDR-H3 paratopes. This enables to primarily sample this diversity of peculiarly long antigen binding sites, whereas other techniques such as B-cell selection strategies or next-generation sequencing (NGS) do not seem to focus on this immune repertoire subset (24). It will be interesting to investigate if the diversity output could be further broadened by combining the herein presented YSD approach with NGS. Another benefit of YSD also relies in its versatility for antibody engineering. In this respect, one of the main applications of this technology is affinity maturation, which can be achieved in multiple ways (36, 38, 43, 48–50). This opens up the possibility to further enhance affinities of binders if needed. Additionally, YSD can be exploited for humanization purposes (51). The bovine IGHV1-7 gene segment only shares about 66% sequence identity with human V gene segments. This is also in the same range for the light chain segment (VLλ30) and human counterparts. Due to this foreign nature, immunogenicity can be anticipated when administered into humans. Hence, humanization of the V gene segments would be required in order to develop this type of molecules for therapeutic purposes. Studies addressing this issue are currently underway. Eventually, even after V gene humanization, the non-human composition (sequence-wise and structural) of stalk-knob architectures might be recognized as foreign by the human adaptive immune system.

In addition to sequence conserved CDRs (besides CDR-H3), we also utilized a fixed light chain for library construction and for the generation of all chimeric IgG-like antibodies. Still, we were able to identify variants binding highly specific and with high affinities to EGFR-positive cells. This might open up engineering possibilities when it comes to bi- and multifunctional IgG-like antibodies (52–54). Besides heavy chain heterodimerization that can be forced using different technologies, such as knob-into-holes and others (55, 56), a typical issue of asymmetric bispecifics relies in specific pairing of a given heavy chain to its cognate light chain. A very elegant approach to circumvent this problem is the generation of the so-called “common light chain” bispecifics, i.e., bispecific antibodies in which both paratopes share the identical light chain (57, 58). Since ultralong CDR-H3 antibodies usually use a single germline VL gene (V30), which typically is relatively invariant with respect to sequence diversity (22), the bovine immunoglobulin repertoire might be considered as a source of almost common light chain entities. Ultimately, further studies need to be conducted to explore the versatility of bovine ultralong CDR-H3 antibodies in terms of bi- and multispecific antibody engineering.

To the best of our knowledge, we generated in the present work for the first time bovine ultralong CDR-H3 paratopes against a receptor tyrosine kinase with relevance in cancer disease (59, 60). EGFR expression is commonly altered in several types of cancer by means of its overexpression, gene amplification, or mutation (61). Monoclonal antibody therapy is one treatment option for patients suffering from EGFR-related tumor burden (62). As of now, three EGFR-targeting antibody therapeutics have been approved for therapy, and a plethora of molecules is currently investigated in clinical trials (1). We were able to demonstrate that by the combination of cattle immunization with YSD, antibodies can be obtained targeting several different epitopes on EGFR, as demonstrated by domain mapping. Interestingly, none of the isolated binders competed with therapeutic antibody Cetuximab or Matuzumab (which has been assessed in clinical trials) for binding to EGFR. It is tempting to speculate that owing to the unique composition of the antigen-binding site of ultralong CDR-H3 antibodies, this unconventional immunoglobulin is predisposed to address a unique epitope space potentially cryptic to classical IgGs. In this regard, Stanfield and colleagues were able to show that a broadly neutralizing anti-HIV bovine ultralong CDR-H3 antibody targets an epitope on the gp120 CD4 receptor binding site (22), typically recessed for conventional antibodies (23). Interestingly, when grafted onto a human IgG1 backbone, all generated chimeric antibody variants in this study were capable of mediating significant ADCC in a target-dependent manner. Some of the generated molecules were almost as potent and efficacious as therapeutic modality Cetuximab in triggering NK cell-mediated lysis of EGFR-overexpressing tumor cell line A431. Moreover, besides 60D01 IgG and 60F06 IgG, all ultralong CDR-H3 antibodies significantly inhibited EGFR-dependent downstream signaling, as demonstrated by analyzing AKT phosphorylation. This is interesting, since none of the antibodies competed with EGF for binding to EGFR. Reasons for inhibiting EGFR-dependent signaling might therefore rely on hindering EGFR domain rearrangement, i.e., in trapping EGFR in the “tethered” conformation (63) or by simply inhibiting receptor dimerization. Macpherson and colleagues, who generated knob domains against complement protein C5 (24), were able to unveil an allosteric modulation of C5 by most isolated knob architectures (25). Further in-depth studies would be needed to reveal the exact mode of action of the herein generated molecules targeting EGFR. Essentially, our findings support the notion that ultralong CDR-H3 paratopes might be versatile for biotechnological and biomedical applications such as the modulation of antitumor responses.

Furthermore, within this work, we scrutinized whether ultralong CDR-H3-derived knob regions can be engineered in a sense that these disulfide-rich architectures function as autonomous paratopes without the stalk region and the antibody scaffold as knob-Fc fusions. Such small Fc fusion proteins can be envisioned to be advantageous for treating solid tumors due to enhanced tissue penetration (64). This was accomplished by simply grafting the knob architectures including residues at the transition between the knob and the stalk region onto the hinge-Fc region of a human IgG1 backbone. Most of the resulting Knobbodies displayed tendencies towards aggregation, clearly disqualifying these molecules for further consideration. Apparently, this would suggest that the antibody scaffold including the stalk region might have a stabilizing effect for the knob region (12, 14). Macpherson et al., however, were able to chemically synthesize solitary knob architectures by solid-phase peptide synthesis, proving that the antibody scaffold including the stalk region is not a prerequisite for the knob structure in order to function properly (65). This clearly paves the way for a multitude of engineering options. In this regard, the authors elegantly demonstrated that the knob structure can be modified, e.g., by the incorporation of non-natural amino acids or by head-to-tail cyclization. In line with these findings, in the present study, two knob-Fc fusions sharing a high-sequence identity showed quite acceptable biophysical properties. For those molecules, a slight to moderate decline in functionalities was observed, indicating that the antibody scaffold including the stalk might fine-tune the spatial orientation of the actual antigen-binding site when the knob structure is placed onto a Fc region. Nevertheless, for both Knobbodies, the main biochemical and functional properties such as specificities, high-affinity binding, and ADCC capabilities, and antagonism were largely retained. In terms of ADCC capacities, we noticed moderately impeded potencies and efficacies. This can be partially explained by slightly reduced affinities of generated Knobbodies. In addition, multiple parameters impact the formation of the immunological synapse, including epitope location (membrane proximal vs. membrane distal) and the molecular format (66). It is obvious that extracting the solitary knob domain from the antibody scaffold tremendously tampers the antibody format. Notwithstanding, this demonstrates that for a subset of bovine ultralong CDR-H3 antibodies, the knob region can function as a paratope independently from the antibody framework as Fc fusion protein without excessive engineering efforts. It will be interesting to investigate whether Knob-Fc fusions with adequate biophysical properties can be engineered in a “plug-and-play.” This kind of optimization would be required for Knobbodies to be more broadly applicable, e.g., on a platform basis for the generation of bi- and multispecific antibody constructs as already shown for single domain antibodies from camelids (66–69) and sharks (4, 70, 71).

## Data Availability Statement

The datasets presented in this study can be found in online repositories. The names of the repository/repositories and accession number(s) can be found in the article/**Supplementary Material**.

## Ethics Statement

The animal study was reviewed and approved by Niedersächsisches Landesamt für Verbraucherschutz und Lebensmittelsicherheit (LAVES), Dezernat 33—Tierschutzdienst, number 33.19-42502-05-17A210.

## Author Contributions

SZ, HK, and LP conceived and designed the majority of experiments. LP, PA, DK, SC, and JH performed experiments. LP, SZ, and HK analyzed the data. SZ and LP wrote the manuscript. SK, LT, DY, and VS gave scientific advice. All authors contributed to the article and approved the submitted version.

## Conflict of Interest

Authors LP, DK, PA, SK, DY, LT and SZ were employed by company Merck Healthcare KGaA. VS is a co-founder of several companies working on cattle-derived antibodies. VS has filed several patents in this field.

The remaining authors declare that the research was conducted in the absence of any commercial or financial relationships that could be construed as a potential conflict of interest.

## Publisher’s Note

All claims expressed in this article are solely those of the authors and do not necessarily represent those of their affiliated organizations, or those of the publisher, the editors and the reviewers. Any product that may be evaluated in this article, or claim that may be made by its manufacturer, is not guaranteed or endorsed by the publisher.
