# Supplementary material for: Milking the Cow: Cattle-Derived Chimeric Ultralong CDR-H3 Antibodies and Their Engineered CDR-H3-Only Knobbody Counterparts Targeting Epidermal Growth Factor Receptor Elicit Potent NK Cell-Mediated Cytotoxicity
Source: Front Immunol. 2021 Oct 25;12:742418. doi: 10.3389/fimmu.2021.742418 (PMC8573386; doi:10.3389/fimmu.2021.742418)
Supplement: Supplementary file 1 [file DataSheet_1.docx]

Supplementary Material

## Supplementary Figures


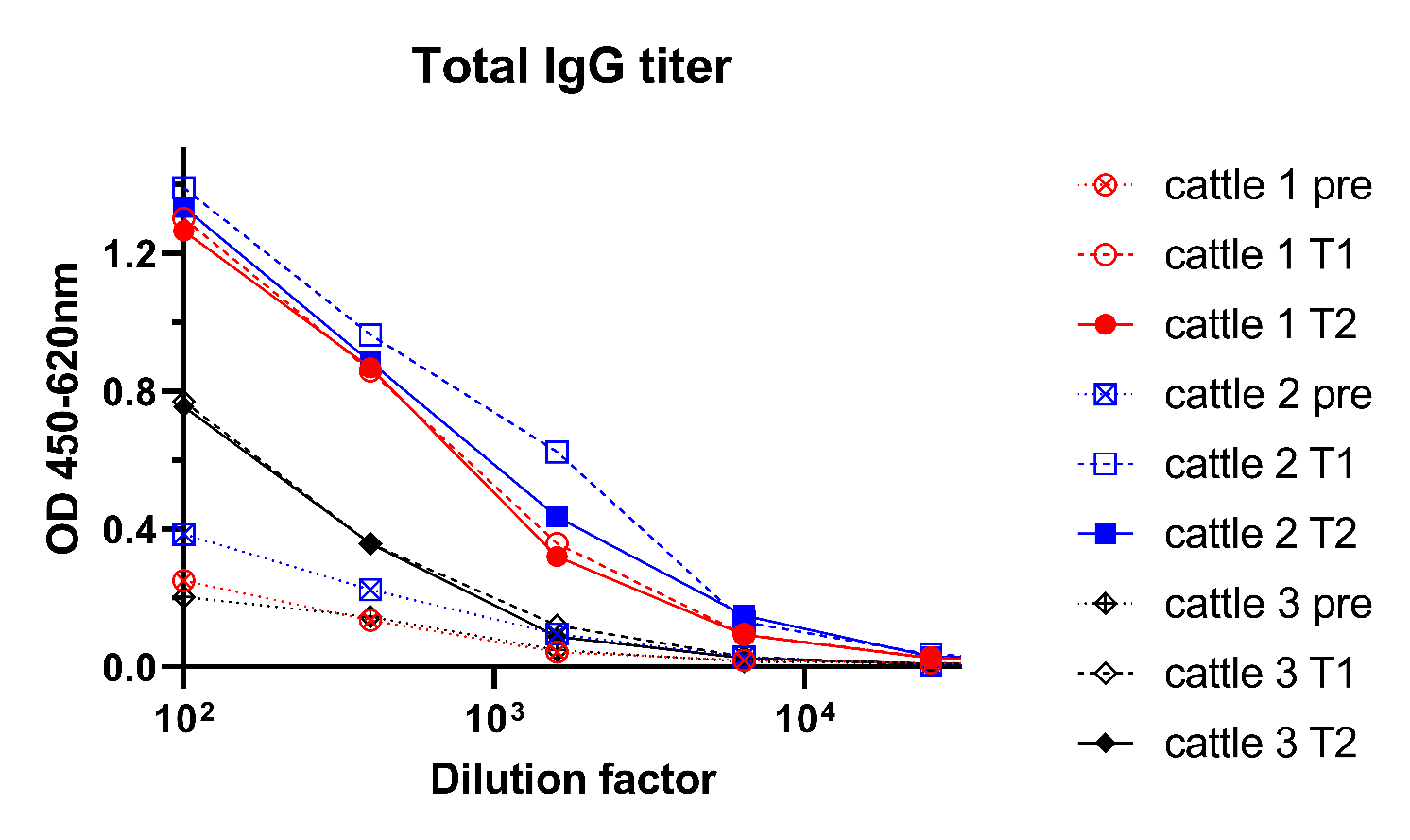


**Supplementary Figure 1.** Immunization of cattle resulted in a significant IgG immune response. Determination of EGFR-specific IgG titer before and during immunization procedure. EGFR was plated followed by incubation with serum in different dilutions and detection via an anti-bovine IgG (H&L) HRP conjugate. Pre, serum before first injection; T1, serum collected on day 52; T2, serum collected on day 80.


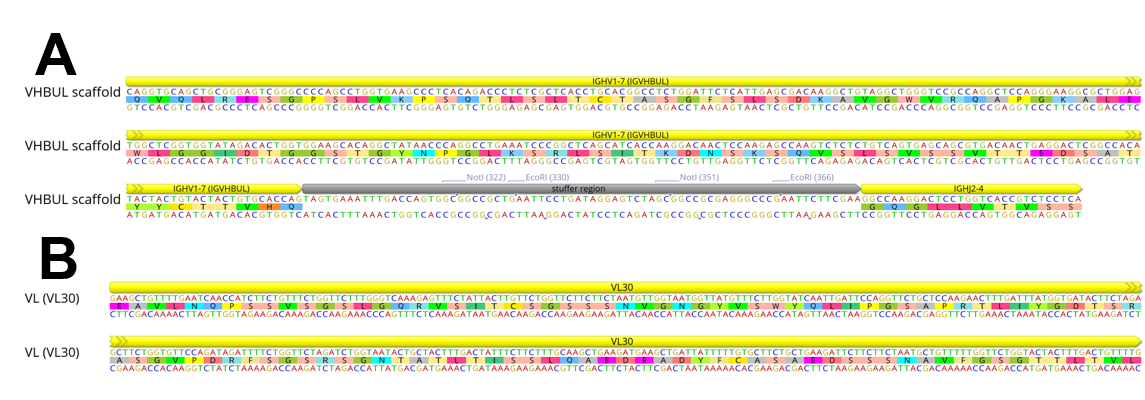


**Supplementary Figure 2.** Variable domain sequences used in this study. (A) VHBul scaffold sequence of bovine IGHV1-7 is given, followed by the stuffer sequence that replaced CDR-H3 and IGHJ2-4 segment. Of note, the stuffer sequence contained stop codons in every possible reading frame as well as two NotI and EcoRI cleavage sites in order to prepare the plasmid for gap repair cloning. Scaffold sequence was fused in frame with human IgG1 constant region CH1 as well as AGA2P. (B) Sequence of bovine variable domain VL30 that was fused in frame to human constant region CLλ. Figure generated with Geneious Prime® v2021.1.1.


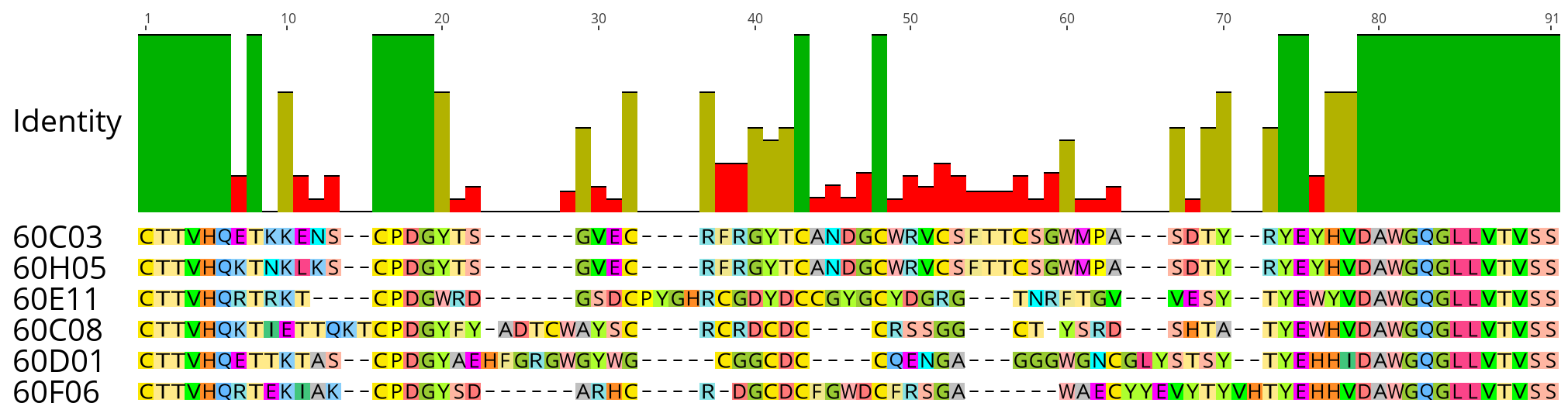


**Supplementary Figure 3.** CDR-H3 sequence alignment of EGFR-specific ultralong chimeric antibodies generated in this study. Sequence of IGHJ2-4 is included as well as sequence names. The six clones belong to five distinct groups of clonotypes out of eight groups that were identified after library selection and sequencing. Alignment generated with Geneious Prime® v2021.1.1.


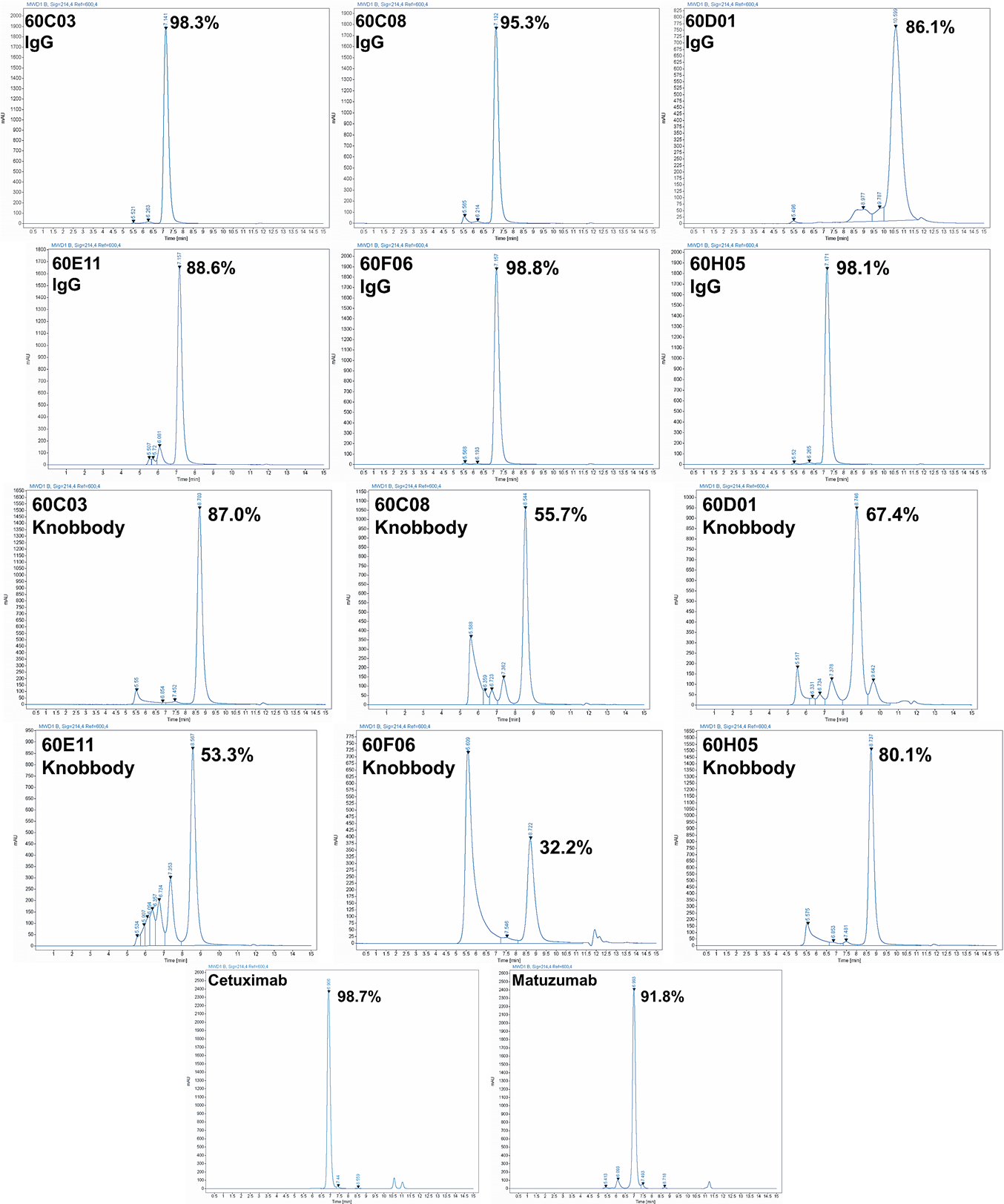


**Supplementary Figure 4.** Analytical size exclusion chromatography profiles of all molecules six antigen-specific chimeric cattle-derived ultralong CDR-H3 IgG antibodies and their Knobbody counterparts as well as Cetuximab and Matuzumab. Of note, 60D01 shows an elution profile later than expected presumably due to interaction with the column matrix.

**
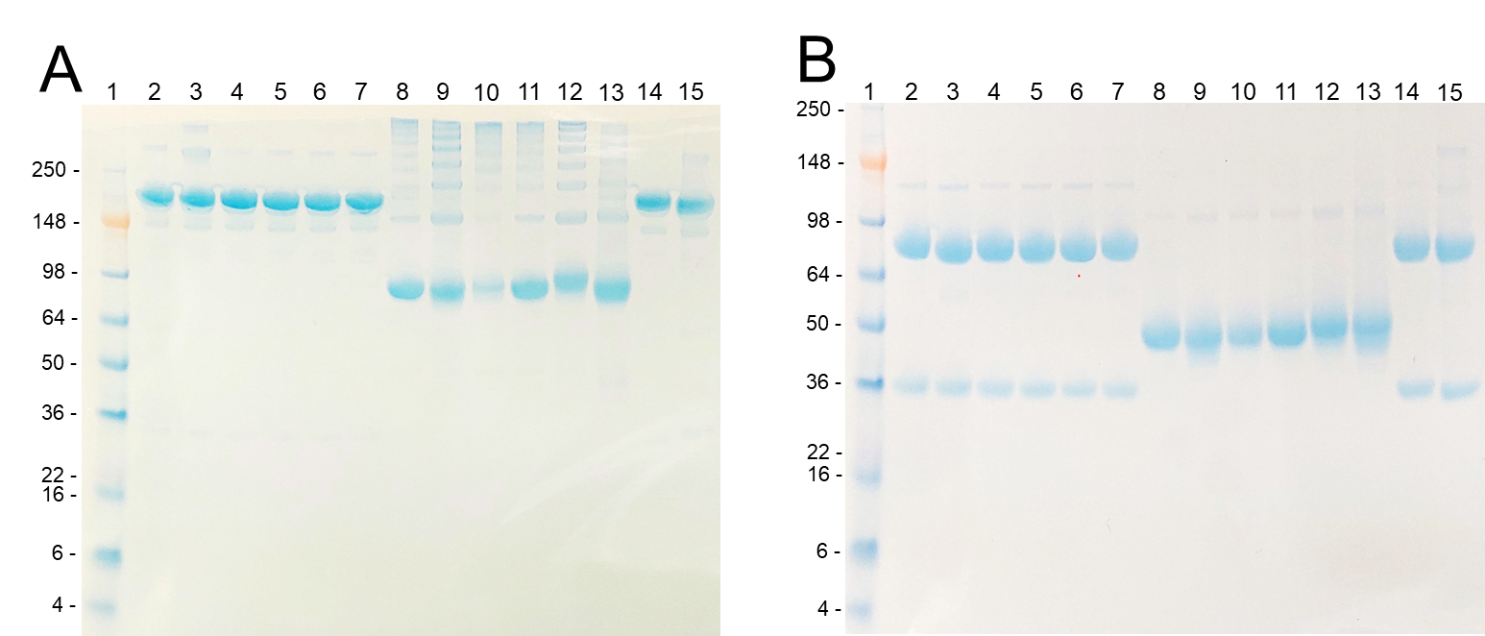
**

**Supplementary Figure 5.** SDS-PAGE analysis of chimeric bovine ultralong CDR-H3 molecules (5 µg) under A) non-reducing or B) reducing conditions. Proteins were separated on a 4-12% Bis-Tris Gel and stained with and InstantBlue® Coomassie Protein Stain. Lane 1: protein ladder; lane 2: 60C03 IgG; lane 3: 60E11 IgG; lane 4: 60F06 IgG; lane 5: 60H05 IgG; lane 6: 60C08 IgG; lane 7: 60D01 IgG; lane 8: 60C03 Knobbody; lane 9: 60E11 Knobbody; lane 10: 60F06 Knobbody; lane 11: 60H05 Knobbody; lane 12: 60C08 Knobbody; lane 13: 60D01 Knobbody; lane 14: Cetuximab; lane 15: Matuzumab.


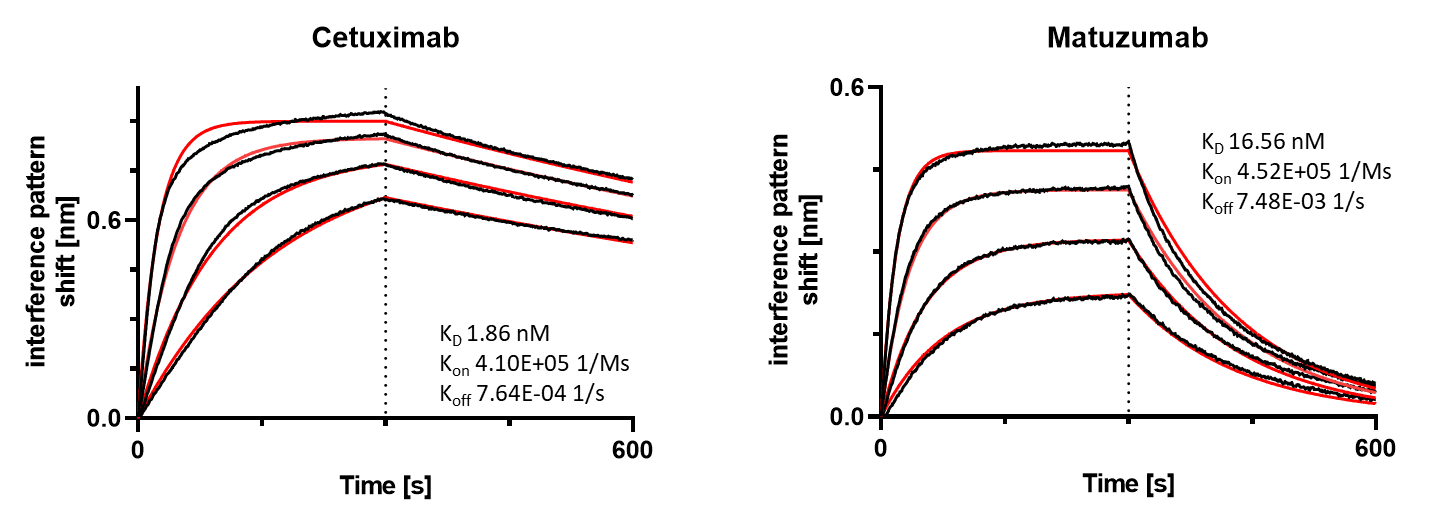


**Supplementary Figure 6**. Kinetic measurements of Cetuximab and Matuzumab against recombinant human EGFR extracellular portion. IgGs were loaded onto sensor tips. After sensor rinsing, antigen binding was conducted at different concentrations (100 nM, 50 nM, 25 nM and 12.5 nM) for 300 s, followed by a dissociation step in kinetics buffer for 300 s.


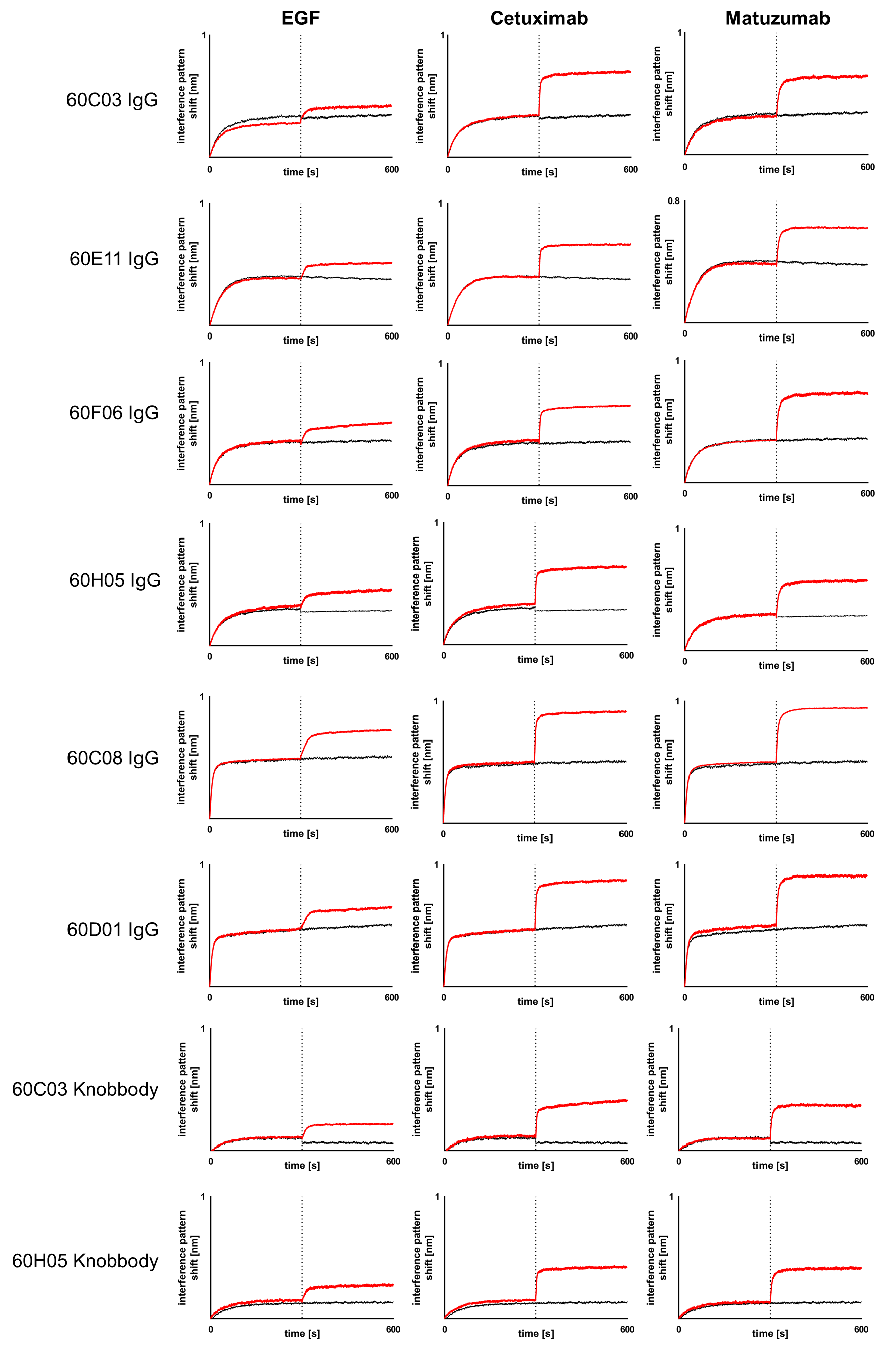


**Supplementary Figure 7.** Non-competitive binding of generated chimeric ultralong CDR-H3 IgGs and Knobbodies to EGFR with natural EGF-Fc fusion, therapeutic entities Cetuximab and Matuzumab as determined by BLI. Recombinant human extracellular domain of EGFR was loaded onto sensor tips, followed by IgG or Knobbody association at a concentration of 100 nM for 300 s. Subsequently, a second association was performed using (at 100 nM for 300 s in presence of 100 nM first analyte) EGF-Fc, Cetuximab or Matuzumab.


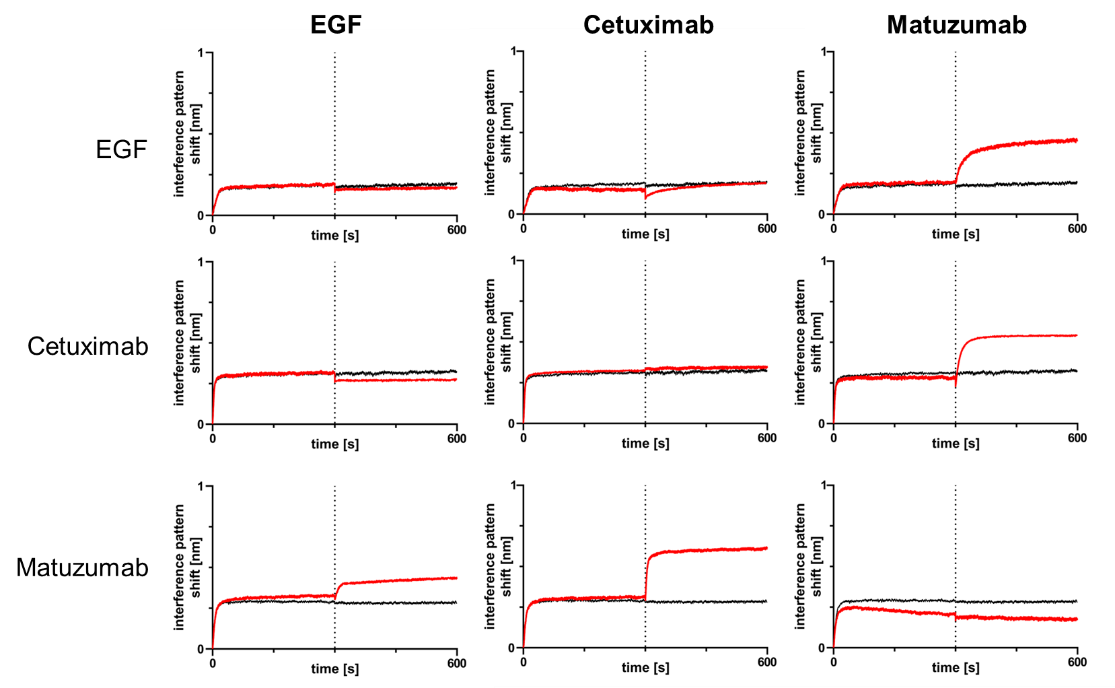


**Supplementary Figure 8.** Non-competitive and competitive binding properties of EGF-Fc fusion, Cetuximab and Matuzumab to EGFR as determined by BLI. Recombinant human extracellular domain of EGFR was loaded onto sensor tips, followed by association of EGF-Fc (top), Cetuximab (middle) or Matuzumab (bottom) at a concentration of 100 nM for 300 s. Subsequently, a second association was performed (in presence of 100 nM first analyte) at 100 nM for 300 s using EGF-Fc, Cetuximab or Matuzumab.

**
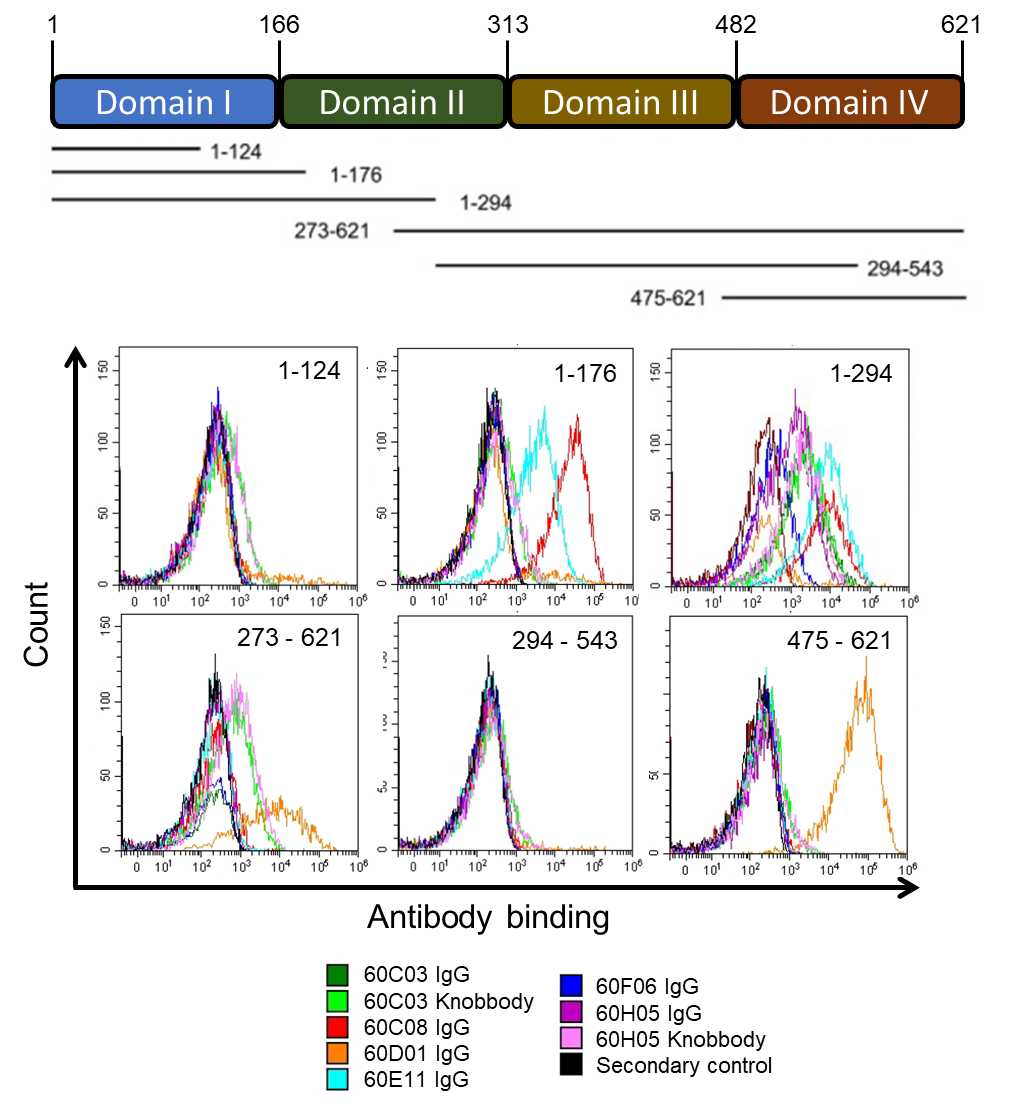
**

**Supplementary Figure 9.** Epitope analysis of chimeric ultralong CDR-H3 antibodies and Knobbodies. Schematic representation of extracellular region of EGFR indicating different subdomains as well as corresponding fragments displayed by YSD (top). Binding of antibodies and Knobbodies to yeast cells displaying different truncated versions of EGFR as determined by flow cytometry (bottom).


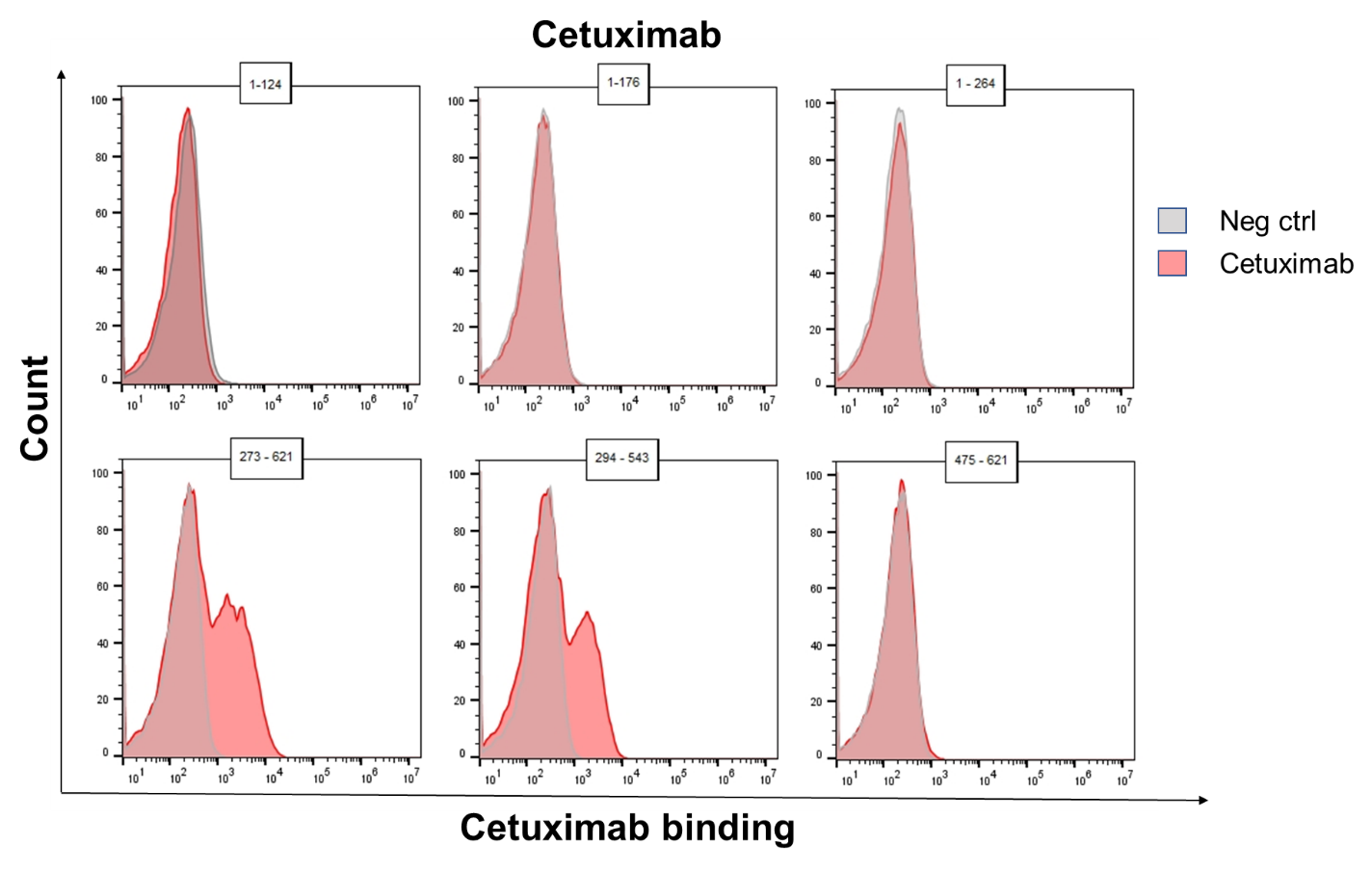


**Supplementary Figure 10.** Epitope analysis of Cetuximab. Binding of Cetuximab to yeast cells displaying different truncated versions of EGFR as determined by flow cytometry.


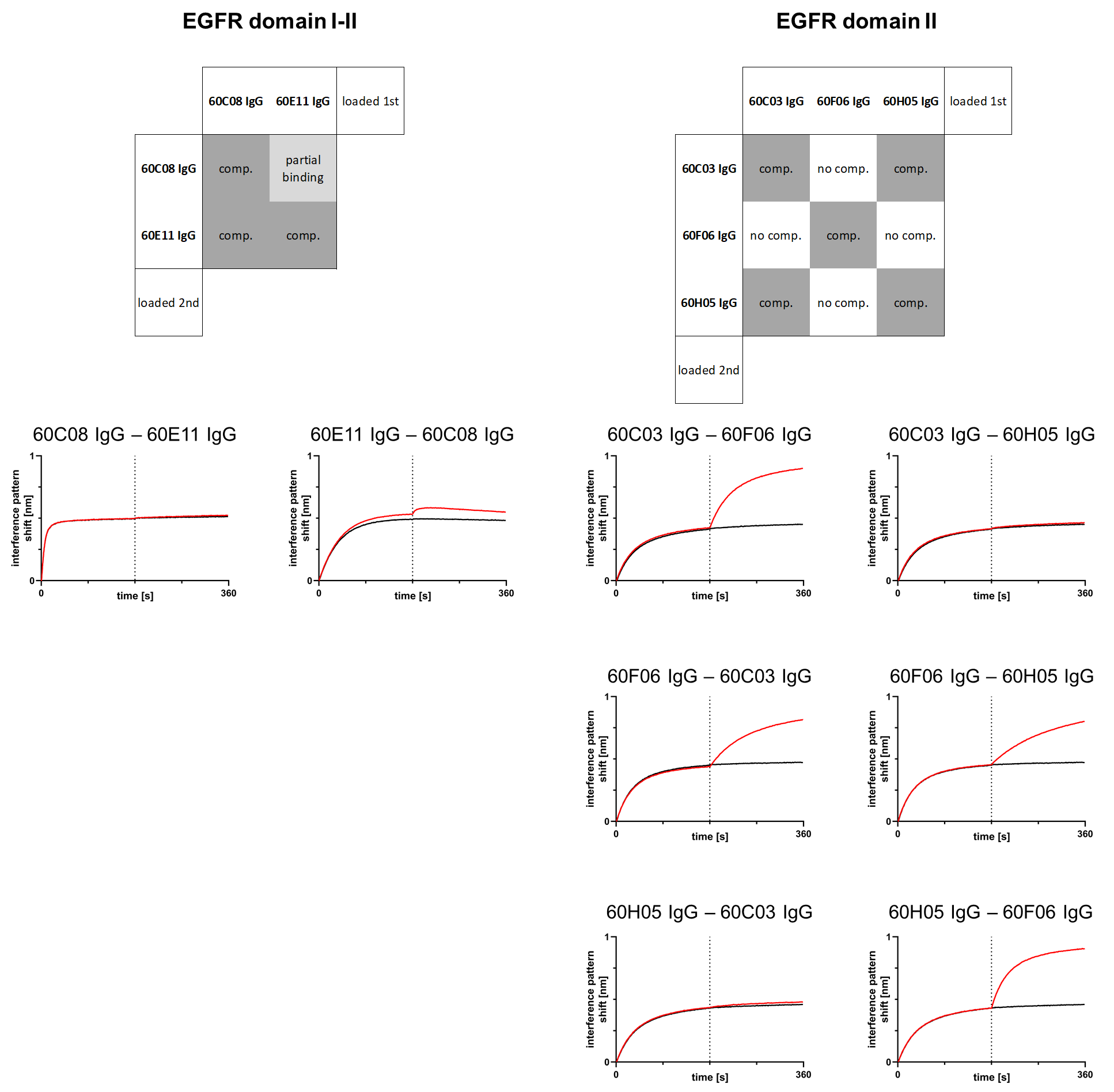


**Supplementary Figure 11.** BLI based competition assays between cattle derived IgG molecules targeting the same subdomain on EGFR. 60C08 IgG and 60E11both bind to yeast-displayed fragments of EGFR corresponding to the interface region between EGFR domain I and II. BLI is measurements indicate an overlapping epitope. 60C03 IgG 60F06 IgG and 60H05 IgG were mapped to EGFR domain II. 60C03 IgG and 60H05 IgG show competitive binding, whereas 60F06 does not compete with 60C03 IgG or 50H05 IgG for binding to EGFR. Recombinant human extracellular domain of EGFR was loaded onto sensor tips, followed by association of the indicated cattle derived IgG at a concentration of 100 nM for 300 s. Subsequently, a second association was performed using another ultralong CDR-H3 antibody (in presence of 100 nM first analyte) at 100 nM for 300 s.


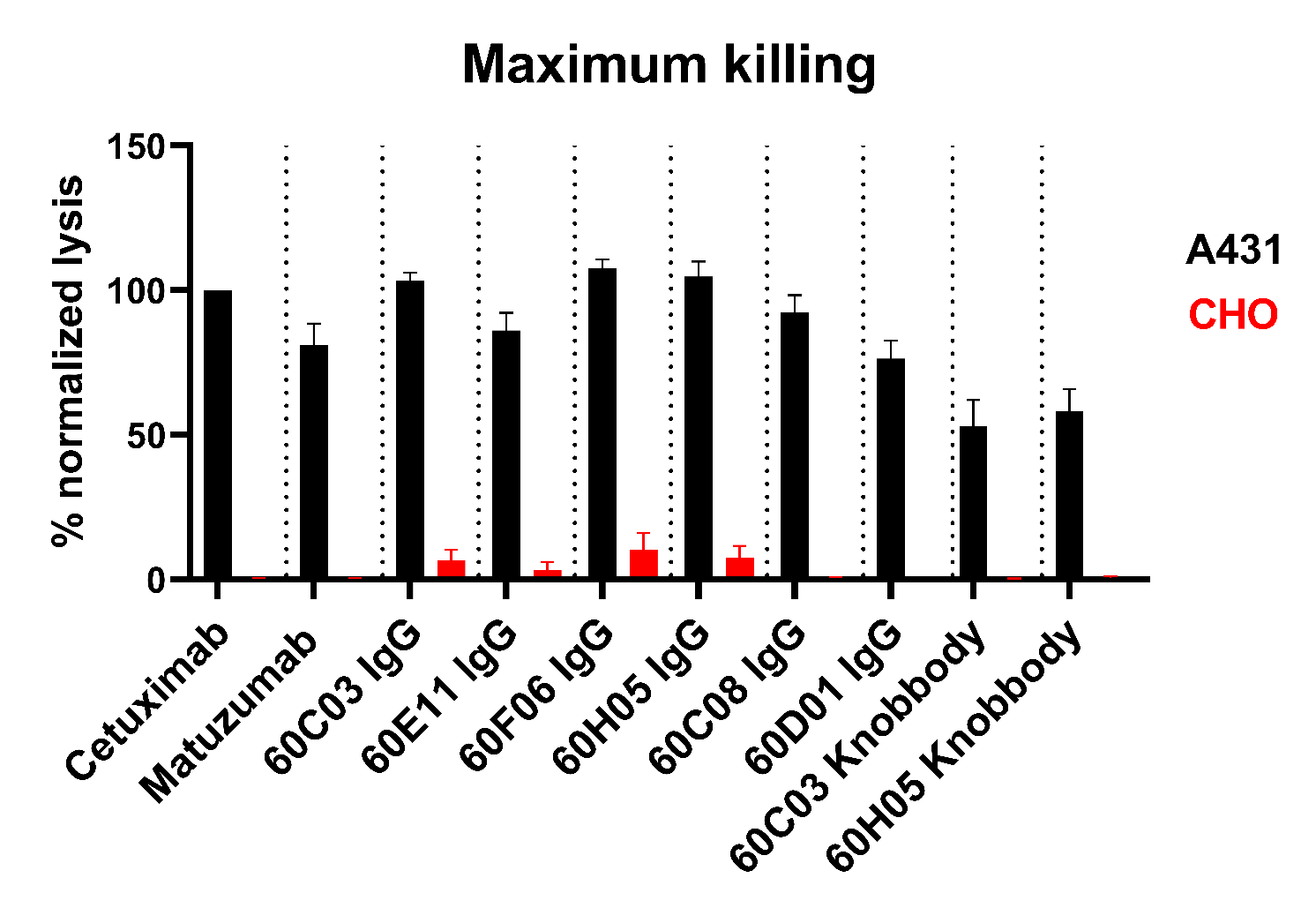


**Supplementary Figure 12.** Specificity of cytotoxic activity of generated chimeric ultralong CDR-H3 antibodies. Fluorescence-microscopy based killing assay with EGFR-positive A431 (black) and EGFR-negative ExpiCHO^TM^ (red) target cells as well as NK effector cells derived from PBMCs at an E:T ratio of 5:1. Analysis of maximum target cell killing (at a concentration of 50 nM for A431 and 500 nM for ExpiCHO^TM^) via NK-mediated ADCC. Comparison of six cow-derived anti-EGFR IgGs and two knobbodies with reference molecules Cetuximab and Matuzumab. Data was normalized to allow for comparison of independent experiments. Graphs show normalized means ± SEM of n = 8 (except n = 4 for 60D01 IgG) different healthy donors.


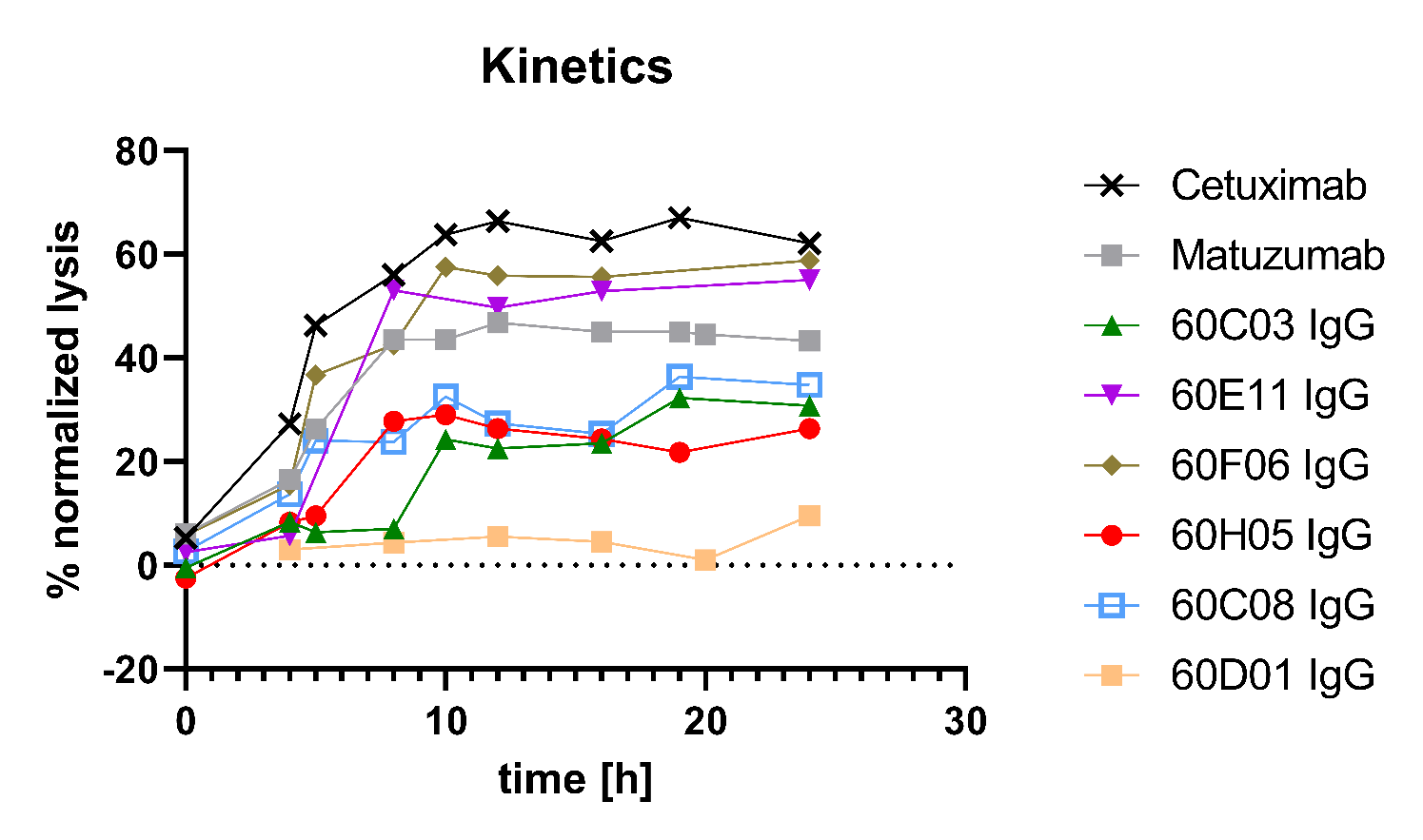


**Supplementary Figure** **13.** Cytotoxic kinetics of generated chimeric ultralong CDR-H3 antibodies. Fluorescence-microscopy based killing assay using EGFR-positive A431 target cells and PBMC-purified NK effector cells at an E:T ratio of 5:1. Analysis of time-dependent target cell killing via NK cell-mediated ADCC at 5 pM sample concentration. Cetuximab (black) and Matuzumab (grey) were included. Data was normalized to maximum killing of Cetuximab at 50 nM after 24 h to allow comparison of the independent experiments. Graphs show normalized means of n = 8 (except for 60D01 IgG, 60C03 Knobbody and 60H05 Knobbody; n = 4) different healthy donors.


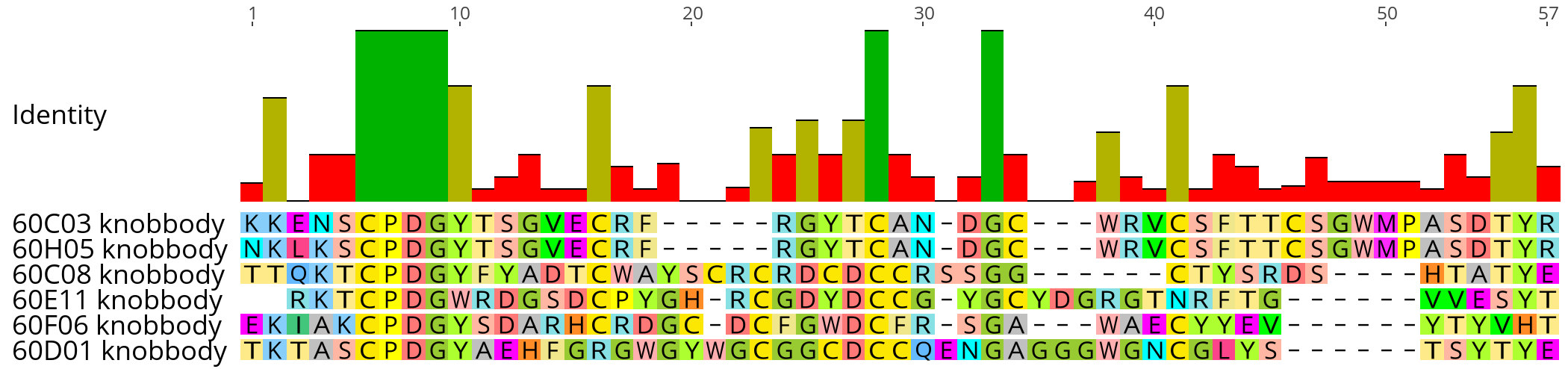


**Supplementary Figure 14.** Sequence alignment of knob architectures as grafted onto the hinge region and Fc part of human IgG1. Of note, one Cys in the hinge region was replaced by Ser. Consequently, the hinge region started with the sequence EPKSS. Alignment generated with Geneious Prime® v2021.1.1.

##
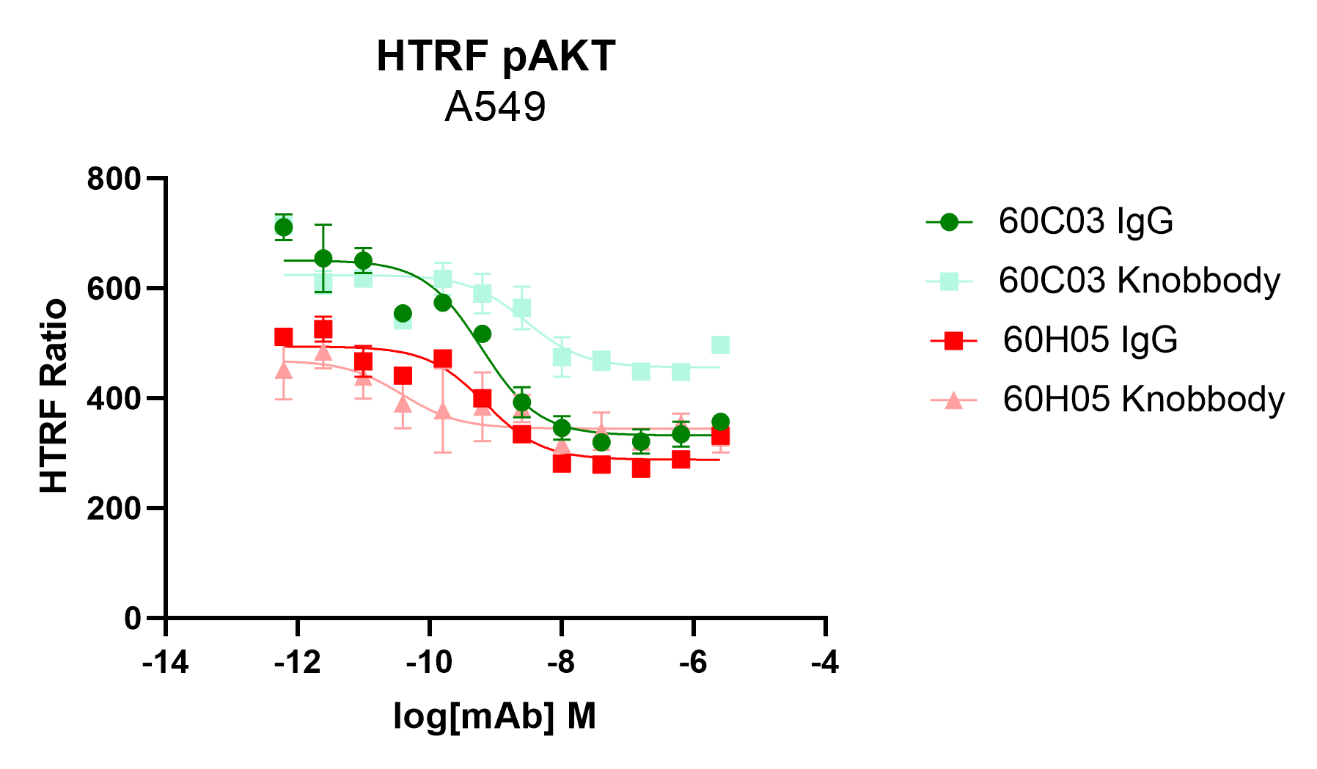


**Supplementary Figure 15.** Inhibition of EGFR-dependent AKT phosphorylation of Knobbodies in direct comparison to their parental IgG counterparts. EGFR positive A549 cells were incubated with the respective antibody derivatives in different concentrations starting from 2.6 µM to 0.62 pM in a four-fold dilution series. Subsequently, EGFR mediated downstream signaling (at a concentration of 20 ng/ml) was monitored by quantitative determination of phospho AKT. All samples were measured in duplicates. HTRF ratio, homogeneous time-resolved fluorescence ratio was calculated according to the manufacturer’s instructions as follows: Acceptor (665 nm) / Donor (620 nm) * 10'000.

## Supplementary Tables

**Supplementary Table 1.** List of primers utilized for specific amplification of bovine ultralong CDR-H3 regions as well as VLλ30.

| **Name** | **Amino acid sequence (*N*-term to *C-*term)** | **Sequence (5’ to 3’)** |
| --- | --- | --- |
| oVHBULL1_CDR3_GR_up | SSVTTEDSATYYCTTV | AGCAGCGTGACAACTGAGGACTCGGCCACATACTACTGTACTACTGTG |
| oVHBULL2_CDR3_GR_up | SSVTTEDSATYYCTTVH | AGCAGCGTGACAACTGAGGACTCGGCCACATACTACTGTACTACTGTGCAC |
| oVHBULL4_CDR3_GR_up | SSVTTEDSATYYCTTVHQ | AGCAGCGTGACAACTGAGGACTCGGCCACATACTACTGTACTACTGTGCACCAG |
| VHBULL5_CDR3_GR_lo | WGQGLLVTVSSASTKG | GCCCTTGGTACTAGCTGAGGAGACGGTGACCAGGAGTCCTTGGCCCCA |
| VHBULL6_CDR3_GR_lo | DAWGQGLLVTVSSASTKG | GCCCTTGGTACTAGCTGAGGAGACGGTGACCAGGAGTCCTTGGCCCCAGGCATC |
| VL30_GR_up | IAAKEEGVQLDKREAVLNQPREQSDRRGGLRLTQNGQSS | CATTGCTGCTAAAGAAGAAGGGGTACAACTCGATAAAAGAGAAGCCGTTTTGAATCAACC |
| VL30_GR_lo | TTLTVLGQPKAAPSVTLFP | GCGGGAACAGAGTGACCGAAGGGGCGGCCTTCGGCTGACCCAGAACGGTCAAAGTAGTAC |

**Supplementary Table 2.** BLI based affinity measurements of all EGFR-specific molecules utilized in this study

| Name | KD (M) | KD Error | kon(1/Ms) | kon Error | kdis(1/s) | kdis Error | Full X^2 | Full R^2 |
| --- | --- | --- | --- | --- | --- | --- | --- | --- |
| Cetuximab | 1.862E-09 | 1.287E-11 | 4.10E+05 | 1513 | 7.64E-04 | 4.47E-06 | 0.4223 | 0.9951 |
| Matuzumab | 1.656E-08 | 1.072E-10 | 4.52E+05 | 2770 | 7.48E-03 | 1.55E-05 | 0.1385 | 0.9972 |
| IgG 60C03 | 1.371E-08 | 5.561E-11 | 1.43E+05 | 484.5 | 1.96E-03 | 4.39E-06 | 0.0463 | 0.9986 |
| IgG 60E11 | 4.103E-09 | 2.814E-11 | 5.42E+05 | 3163 | 2.22E-03 | 8E-06 | 0.0122 | 0.9907 |
| IgG 60F06 | 7.385E-09 | 3.094E-11 | 1.18E+05 | 298.3 | 8.70E-04 | 2.9E-06 | 0.0403 | 0.9993 |
| IgG 60H05 | 2.79E-08 | 2.164E-10 | 1.14E+05 | 810.5 | 3.19E-03 | 1E-05 | 0.0828 | 0.9965 |
| Knobbody 60C03 | 3.661E-08 | 4.489E-10 | 6.10E+04 | 684.2 | 2.23E-03 | 1.1E-05 | 0.041 | 0.9957 |
| Knobbody 60H05 | 3.537E-08 | 1.963E-10 | 5.35E+04 | 271 | 1.89E-03 | 4.3E-06 | 0.0105 | 0.9992 |
| IgG_60C08 | 4.778E-08 | 6.625E-10 | 5.97E+05 | 7880 | 2.85E-02 | 0.000122 | 0.0908 | 0.9975 |
| IgG_60D01 | 1.20E-07 | 5.253E-09 | 6.24E+05 | 25960 | 7.49E-02 | 0.001018 | 0.0859 | 0.9905 |
